# Chronic stress dysregulates the Hippo/YAP/14-3-3η pathway and induces mitochondrial damage in basolateral amygdala in a mouse model of depression

**DOI:** 10.7150/thno.92676

**Published:** 2024-06-11

**Authors:** Yan Zhao, Yulong Chen, Chihua Guo, Pingping Li, Zhao Cheng, Lei Zheng, Baoyong Sha, Hao Xu, Xingli Su, Yunpeng Wang

**Affiliations:** 1 Department of Basic Medicine Science & Shaanxi Key Laboratory of Brain Disorders, Xi'an Medical University, Xi'an, Shaanxi 710021, China.; 2 Institute of Basic and Translational Medicine, Xi'an Medical University, Xi'an, Shannxi 710021, China.; 3 Center for Translational Medicine, The First Affiliated Hospital of Xi'an Jiaotong University, Xi'an, Shaanxi 710061, China.; 4 School of Pharmacy, Xi'an Medical University, Xi'an, Shaanxi 710021, China.; 5 Department of Psychiatry and Center for Brain Science, The First Affiliated Hospital of Xi'an Jiaotong University, Xi'an, Shaanxi 710061, China.; 6 Lead contact.

**Keywords:** depression, YAP, Hippo pathway, mitochondria, basolateral amygdala

## Abstract

**Rationale**: Recent evidence highlights the pivotal role of mitochondrial dysfunction in mood disorders, but the mechanism involved remains unclear. We studied whether the Hippo/YAP/14-3-3η signaling pathway mediates mitochondrial abnormalities that result in the onset of major depressive disorder (MDD) in a mouse model.

**Methods**: The ROC algorithm was used to identify a subpopulation of mice that were exposed to chronic unpredictable mild stress (CUMS) and exhibited the most prominent depressive phenotype (Dep). Electron microscopy, biochemical assays, quantitative PCR, and immunoblotting were used to evaluate synaptic and mitochondrial changes in the basolateral amygdala (BLA). RNA sequencing was used to explore changes in the Hippo pathway and downstream target genes. *In vitro* pharmacological inhibition and immunoprecipitation was used to confirm YAP/14-3-3η interaction and its role in neuronal mitochondrial dysfunction. We used virus-mediated gene overexpression and knockout in YAP transgenic mice to verify the regulatory effect of the Hippo/YAP/14-3-3η pathway on depressive-like behavior.

**Results**: Transcriptomic data identified a large number of genes and signaling pathways that were specifically altered from the BLA of Dep mice. Dep mice showed notable synaptic impairment in BLA neurons, as well as mitochondrial damage characterized by abnormal mitochondrial morphology, compromised function, impaired biogenesis, and alterations in mitochondrial marker proteins. The Hippo signaling pathway was activated in Dep mice during CUMS, and the transcriptional regulatory activity of YAP was suppressed by phosphorylation of its Ser127 site. 14-3-3η was identified as an important co-regulatory factor of the Hippo/YAP pathway, as it can respond to chronic stress and regulate cytoplasmic retention of YAP. Importantly, the integrated Hippo/YAP/14-3-3η pathway mediated neuronal mitochondrial dysfunction and depressive behavior in Dep mice.

**Conclusion**: The integrated Hippo/YAP/14-3-3η pathway in the BLA neuron is critical in mediating depressive-like behaviors in mice, suggesting a causal role for this pathway in susceptibility to chronic stress-induced depression. This pathway therefore may present a therapeutic target against mitochondrial dysfunction and synaptic impairment in MDD.

## Introduction

Major depressive disorder (MDD) is characterized by persistent depressed mood, loss of interest and pleasure in previously enjoyed activities, recurrent thoughts of death, and physical and cognitive symptoms 1. According to the World Health Organization (WHO 2021), MDD is the leading cause of global mental and physical disability, one of the main contributors to the global burden of disease, with more than 264 million people of all ages currently afflicted 2. Patients with MDD show profound changes in behavior, including sad mood, anhedonia, anxiety, fatigue, psychomotor retardation, and social withdrawal 3. However, the pathophysiology of MDD is complicated and largely unknown due to substantial dysregulation of the molecular and signaling pathways.

External stress is widely recognized as the most crucial precipitating factor for MDD, particularly in individuals with a genetic predisposition. The chronic unpredictable mild stress (CUMS) model is a classic rodent model that replicates the diverse and unpredictable physical and mental irritations that humans experience in their daily lives. CUMS produces behavioral changes that are believed to mirror key symptoms observed in depressed humans, such as anhedonia, anxiety, and despair 4. Although MDD patients exhibit a wide range of combination of symptoms, animals exposed to CUMS are often considered a uniform group. Therefore, it is crucial to develop a new approach that recognizes the heterogeneity of stress-induced behavioral phenotypes to better dissect the specific neural correlates of the depressive symptom.

The new hypothesis of mood disorders highlights the strong association of neuronal impairment and mitochondrial dysfunction 5. Mitochondria in the brain are pivotal for influencing neural activity, synaptic plasticity, and behavioral adaptation. Mitochondrial dysfunction not only hinders energy requirements but also disrupts neuronal communication and cellular resilience. Importantly, mitochondrial dysfunction has been shown to be crucial in MDD 5, 6, bipolar disorder 7, 8, and other neuropsychiatric diseases 9, 10. The presence of underlying mitochondrial dysfunction in mood disorders can be inferred from various evidences, such as decreased cellular respiration, altered mitochondrial structure, mtDNA mutations, and reduced respiration chain proteins 11, 12. Although several lines of evidence have indicated that antidepressants can alter neuronal energy metabolism and affect the mitochondrial process 13, 14, interventions targeting mitochondrial dysfunction in MDD are currently lacking.

The Hippo pathway is a highly conserved signaling cascade that primarily regulates cell survival, proliferation, apoptosis, as well as mitochondrial function 15. The Hippo pathway primarily outputs signal through the transcriptional coactivator Yes-associated protein (YAP), which is orchestrated with transcription factors such as the TEA-domain family member1 (TEAD1), to regulate the expression of numerous target genes. Nuclear localization and transcriptional activity of YAP are suppressed after phosphorylation by upstream kinases, such as mammalian sterile 20-like kinase 1/2 (Mst1/2) and large tumor suppressor kinases 1/2 (Lats1/2) 16. Although discovered for its role in tumor growth, the Hippo/YAP pathway has recently been emphasized as a key mediator in various neuropsychiatric diseases, including MDD in humans and animal models 17-19. Together, these studies demonstrate that external stress may alter neuronal mitochondrial function by disrupting the Hippo/YAP pathway. Therefore, we used the CUMS mice model to investigate the hypothesis that inhibiting Hippo signaling or increasing YAP nuclear activity ameliorates mitochondrial dysfunction and synaptic impairment in basolateral amygdala neurons (BLA) and strengthens individual resilience to stress.

## Methods

### Animals

Adult C57BL/6J mice (male, 8-10 weeks old) were obtained from the Laboratory Animal Center of Xi'an Jiaotong University (Xi'an, China). Yap1-floxed (Yap1^fl/fl^) mice, which is from C57/BL6J background, was generated by Cyagen Biosciences (KOCMS180105HY1, Cyagen, Suzhou, China). The gRNA to mouse Yap1 gene, the donor vector containing loxP sites, and Cas9 mRNA were co-injected into fertilized mouse eggs to generate targeted conditional knockout offspring. All animals were kept in a temperature- and humidity-controlled environment with a 12/12 h light-dark cycle (lights on at 7:00). All animals were acclimated to housing conditions around 2 weeks (3 mice per cage, food and water *ad libitum*) before any experimental manipulations. In all experiments, age-matched littermates were used. All mice received treatments in the same order. All protocols and procedures used in this study were approved by the Institutional Animal Care and Use Committee of the Xi'an Jiaotong University and conformed to the Guide for the Care and Use of Laboratory Animals published by NIH. All efforts were made to minimize the number of animals used and their suffering.

### CUMS paradigm

The CUMS paradigm was performed as we previously described 20. Mice in the CUMS group were exposed to 8 weeks of consecutive stress, while control mice received only regular handling. Briefly, after 5 days of initial habituation, mice were subjected to various unpredictable mild stressors for a period of 8 weeks. Weekly stress consisted of the following stressors in random order: 45° cage tilting, wet bedding, fasting, water deprivation, swimming in 4 °C cold water, swimming in 40 °C hot water, nip tail, shaking, paired housing and inversion of the light/dark cycle. On average, two different stressors were applied per day. At the end of every week, the body weight and sucrose preference of all mice were assessed. Behavior tests were carried out in the last week. The control group was left unchallenged except for 24 h water deprivation before sucrose preference test. Based on previous evidences 21, as well as our recent report 22 and preliminary experiments, 3 days of repeated stress is sufficient to trigger depressive phenotypes in stress-susceptible mice. Therefore, we used the 3-day social defeat to distinguish the changes in stress resilience in mice.

### Behavioral assays

All behavioral tests were performed in a temperature (20-22 °C) and humidity (40-60%) controlled room. Each animal was allowed 15 min of acclimatization prior to tests. All behaviors were carried out without the experimenter being present in the room. The behavioral equipment was cleaned with 70% ethanol between individual animals. Animal behaviors were recorded and analyzed using Any-maze video tracking software (Stoelting Co., Wood Dale, USA).

#### Sucrose preference test (SPT)

Each animal was provided with two drinking tubes in their home cages during the 24 h training phase. After training, mice were deprived of water for 24 h, then the mice were given the choice to drink from two bottles for 12 h: one was filled with a sucrose solution (1% w/v), and the other was filled with water. The positions of the bottles in the cage were switched after 6 h. Sucrose and water consumptions were recorded before and after the test. Sucrose preference% = (sucrose intake/total intake) × 100%.

#### Elevated plus maze (EPM)

The EPM test consisted of a plus-shaped platform with four 33×6 cm plates connected to a central platform (6×6 cm). Two opposing close arms were enclosed by walls, whereas two open arms were not enclosed. The mice were placed individually in the center of the apparatus for 5 min and the time spent and number of entries in the open arms was recorded to detect the anxiety of the mice.

#### Forced swim test (FST)

Mice were placed in a glass cylinder (height 27 cm, diameter 18 cm, filled with 3.5 liters of water at 24±2 °C) to swim for 6 min, and the immobility time was automatically monitored during the last 4 min of the test. Latency to the first immobilization was also recorded.

#### Novelty suppressed feeding (NSF)

The NSF test was performed in a plastic box (50×50×20 cm). The floor was covered with approximately 2 cm of bedding and the arena was brightly lit (1200 lux). The mice were food restricted for 24 h. At the time of the test, a single pellet of food was placed on a white paper platform positioned in the center of the box. Each animal was placed in a corner of the box. The amount of time to take the first bite was recorded as latency to feed. The consumption of home cage food was measured immediately after the test as a control value.

#### Open-field test (OFT)

A black square arena (45×45×30 cm) was used to examine locomotor activity. Mice were placed in the arena and allowed to explore the apparatus freely for 15 min. The total distance was analyzed by the video tracking software.

### Stereotaxic viral injection

The adeno-associated virus (AAVs) used in this study were constructed and produced by the GeneChem Co. (Shanghai, China) rAAV-CaMKIIa-YAP-eGFP, rAAV-CaMKIIa-eGFP-sh14-3-3η and BrainVTA (Wuhan, China): rAAV-CAMKIIa-eGFP-P2A-Cre, rAAV-VGAT1-CRE-mCherry-WPRE-hGH polyA. During surgery, mice were fixed in a stereotaxic apparatus (RWD Life Science, Shenzhen, China) under isoflurane anesthesia. The coordinates used for BLA injection were: AP -1.4 mm, ML ±3.3 mm, DV -4.6 mm. A volume of 200~300 nL virus was infused bilaterally into the target brain region using a calibrated glass microelectrode attached to a 1 μL Hamilton microsyringe at a flow rate of 30 nL/min. The injector was left in place for an additional 15 min to minimize diffusion through the injector tract. Following surgery, the mice were housed separately for 48 h and then placed back in their home cage.

### Nuclear or cytoplasmic fractionation

Cytoplasmic and nuclear fractionation was performed with the Nuclear and Cytoplasmic Protein Extraction Kit (Beyotime, Shanghai, China) according to the manufacturer's instructions. Protein and RNA from cytoplasmic and nucleus fractionations were analyzed by Western blot and qPCR, respectively.

### Immunoblotting

Mice bilateral BLA were dissected and stored at -80 °C. Protein was extracted from BLA tissue by homogenization with lysis buffer containing phosphatase protease inhibitors. Western blot was performed as previously described 23. The primary antibodies and dilutions used were showed in Supplementary Table S1. Primary antibody incubation overnight at 4 °C was followed by washes in TBST before incubation with the HRP-conjugated secondary antibodies. Immunoreactive bands were detected with an enhanced chemiluminescence kit (Millipore, Billerica, MA, USA). ImageLab software (Bio-Rad Laboratories, Hercules, CA, USA) was used to analyze the band density.

### Immunoprecipitation

HT-22 cells were transfected with Flag-YAP1 plasmid. Cells were then lysed with lysis buffer (50 mM HEPES at pH 7.5, 150 mM NaCl, 1 mM EDTA, 1% NP-40, 10 mM pyrophosphoate, 10 mM glycerophosphate, 50 mM NaF, 1.5 mM Na_3_VO_4_, protease inhibitor cocktail (Roche, US), 1 mM DTT, 1 mM PMSF) and immunoprecipitated with anti-Flag antibody. The immunoprecipitates were washed three times and treated with phosphatase as described before 20. The immunoprecipitated protein was subjected to SDS-PAGE and transferred to PVDF membrane followed by immunoblotting using antibodies against pYAP (Ser127) (1:1000) and 14-3-3η (1:500), respectively.

### Quantitative real-time PCR

Total RNA was extracted using TRIzol reagent after treatment with DNase I (Takara, Japan); RNA was then reverse transcribed using reverse transcriptase (Takara, Japan), and then mature mRNA levels were detected in a Roche 480 Light Cycler or QuantStudio Real-Time PCR System. Relative mRNA expression levels were determined by normalization of target genes to GAPDH as internal control. The primers used are shown in Supplementary Table S2.

### Transmission electron microscopy (TEM)

Mice were perfused intracardially with PBS and fixatives (2% PFA and 2.5% glutaraldehyde in 0.1 M phosphate buffer). Bilateral BLA tissues were removed and post-fixed with 4% PFA and 0.25% glutaraldehyde. The 250 μm coronal sections were then cut with a vibratome (Leica, Mannheim, Germany). The BLA samples were dehydrated with graded ethanol and embedded with low viscosity resin. Ultrathin 70-nm sections were obtained using a Reichert-Jung Ultramicrotome, thin sections were collected on formvar-coated, single-slot copper grids. Ultrathin stained sections were prepared and stained with 2% uranyl acetate and lead citrate for 30 min. Finally, synapse and mitochondria were observed with a JEOL JEM-100SX transmission electron microscope (Hitachi, Ltd., Tokyo, Japan).

### Mitochondrial function assays

#### Mitochondrial membrane potential

We measured the mitochondrial membrane potential (ΔΨm) using JC-1 fluorescence mitochondrial staining assay according to the manufacturer's instructions (Beyotime, Shanghai, China). Cultured HT-22 cells were treated with BDEV or BDEV+NS1619 (40 μM) for 12 h, and then the ΔΨm was determined by measuring changes in JC-1-derived fluorescence from red (J-aggregates) to green (monomeric) using fluorescence microscopy.

#### ATP production

The ATP production levels was measured with an ATP content detection kit (Solarbio, Beijing, China) according to the manufacturer's instructions. Briefly, after behavior tests, BLA were isolated on ice and subsequently lysed in 100 μL of extracting solution. The tissues were crushed by ultrasonic for 1 min on ice. Subsequently, the supernatant (50 μL) was mixed with 50 μL of chloroform and centrifugated (10,000 × *g*) at 4 °C for 3 min. Then transferred the supernatant into a tube and placed on ice for measurement. The absorbance values A1 (0 min) and A2 (3 min) at 340 nm and 37 °C were recorded on a multi-mode microplate detection system (Thermo, MA, USA). ΔA = A2 - A1. ATP production (μmol/g) = 0.625 × ΔA_test_/(ΔA_standard_×W_test_). Relative ATP production = ATP production ^(experiment)^ / ATP production^ (control)^ × 100%.

#### ETC complex activity

The activity of the ETC complex I, II, III, IV, and V and the activity of citrate synthase were measured with the commercial Mitochondrial Complex Activity Kit (Solarbio, Beijing, China) following the manufacturer's instructions. In summary, mitochondrial homogenates were added to the respective reaction buffer. The reaction buffer was prewarmed at 37 °C for 15 min. The absorbance values A1 (0 min) and A2 (1 min) at 550 nm on were measured on a multimode microplate detection system. Mitochondrial complex activity was expressed as nmol/min/mg protein. ΔA1 = A1^test^ - A2^test^. ΔA2 = A1^blank^ - A2^blank^. ΔA = ΔA1 - ΔA2. Complex activity (U/mg prot) = 1099 × ΔA/W_protein_. Relative mitochondrial complex activity = complex activity ^(experiment)^ / complex activity ^(control)^ × 100%. W_protein_: weight of the sample.

#### mtDNA content

The mitochondrial DNA (mtDNA) content in the BLA was determined by qPCR. Total DNA was extracted from tissues using the tissue DNA kit (Omega Biotek, Doraville, GA, USA). Quantitative real-time PCR was performed using gene specific primers and DNA was amplified in a Light Cycler 96 real-time PCR system (Bio-Rad Laboratories, Hercules, CA, USA) using Takara TB Green Premix Ex Taq^TM^ II (Takara Bio, Shiga, Japan). A mitochondrial genome encoded gene *Cox1* and *Cytb* 24 were normalized by simultaneous measurement of the nuclear housekeeping gene *GAPDH*, and relative expression levels between control and treated samples were calculated using the ^ΔΔ^Ct method, as previously described 25.

### TUNEL staining

The frozen brain tissues were embedded with an OCT compound (Leica, Wetzlar, Germany). Tissues were cut into 20 μm thick sections and analyzed with the TUNEL apoptosis assay kit (Beyotime, Shanghai, China) according to the manufacturer's protocol with minor modifications. Briefly, sections were fixed with 4% paraformaldehyde for 20 min at room temperature. Then, they were washed twice for 10 min in PBS and permeabilized in PBS with 0.3% Triton X-100 for 5 min at 37 °C. The sections were then incubated with TUNEL-mixed detection solution at 37 °C in a dark and humid environment for 1 h followed by three 10-min rinses with PBS. Finally, sections were incubated with DAPI staining solution for 5 min at room temperature (Beyotime, Shanghai, China). TUNEL staining was detected with a Cy3 filter. The sections were observed under a fluorescence microscope (Nikon, Chiyoda, Japan) and the results were calculated using Image-ProPlus 6.0.

### Cell culture, transfection, and viral infection

HT-22 mouse hippocampal neuronal cells were obtained from the American Type Culture Collection (ATCC, VA, USA) and were maintained in DMEM medium (Gibco, MD, USA) with 10% FBS, 100 units/mL of penicillin and 100 μg/mL of streptomycin at 37 °C in a humidified atmosphere of 5% CO_2_. All the experiments include a vehicle control group containing 0.1% DMSO. Transfection with Lipofectamine 3000 was performed according to the manufacturer's instructions (L3000-015, Invitrogen, Carlsbad, USA). The pCMV-Flag-YAP1 construct was produced by the GeneChem Co. (Shanghai, China). Short interfering RNA (siRNA) oligonucleotides toward mouse YAP1, 14-3-3η, and negative control siRNA were synthesized by Obio Technology (Shanghai, China). The YAP siRNA sequence (siYAP) was 5′-AGAGATACTTCTTAAATCA-3′. The 14-3-3η siRNA sequence (si14-3-3η) was 5′-CAAGCCTTCGATGATGCTATA-3′. The scrambled sequence (siNC) was 5′-UUCUCCGAACGUGUCACGUTT-3′ or 5′-TTCTCCGAACGTGTCACGT-3′. HT-22 cells were transfected with the respective siRNAs twice in a 24 h interval. Cell lysate was obtained 48 h after transfection. For pan 14-3-3 inhibition, HT-22 cells were treated with BV02 (40 μM; Sigma, St. Louis, USA) or DMSO for 1 h and harvested for future study.

### Immunofluorescence staining

Fresh frozen brain tissue was sectioned at 30 µm and stained using previously described methods 26. In summary, mice were transcardially perfused with 0.01M PBS and 4% paraformaldehyde (PFA) in phosphate buffered saline (PBS). The brains were further postfixed in 4% PFA at 4°C and subsequently stored in 30% sucrose solution in 0.2M PB at 4 °C. The entire brain was serially cut into 30-μm-thick transverse sections with a freezing microtome (Leica, Germany). BLA sections were incubated with primary antibody overnight at 4 °C and then incubated with Cy3-conjugated or FITC-conjugated antibodies. Data were analyzed using ImageJ software.

### Transcriptome analysis

The transcriptome sequencing of the extracted RNA from BLA tissue was performed by Novogene (Beijing, China, https://cn.novogene.com). Briefly, after mRNA enrichment with poly-T oligo-attached magnetic beads, sequencing libraries were generated using the NEBNext Ultra RNA Library Prep Kit for Illumina (NEB, USA) according to the manufacturer's instructions. After quality control analysis using the Agilent 2100 bioanalyzer, barcoded libraries were pooled in equimolar ratios and sequenced on the NovaSeq 6000 platform and 150 bp paired-end reads were generated. The raw data of the Fastq files were quality trimmed, followed by mapping to the mouse reference transcriptome using Hisat2 v2.0.5. Feature Counts v1.5.0-p3 was used to count the reads numbers mapped to each gene. Genes with fewer than 10 reads per sample on average were discarded. Differential expression analysis of two groups was performed using the DESeq2 R package 1.20.0. Genes with a *P*-value < 0.05 found by DESeq2 were assigned as differentially expressed. Gene ontology (GO) and KEGG enrichment analysis of differentially expressed genes was implemented by the clusterProfiler R package, in which the gene length bias was corrected. Heatmaps were generated with the heatmap.2 function.

### Statistical analysis

To determine whether individual animals were positive or negative for a specific behavioral phenotype as measured in SPT, EPM, and FST, we used ROC curves 27. The Youden index was calculated from the ROC curves to identify the optimal cut-off value that gave the lowest false positive rate (FPR) and the highest true positive rate (TPR). Youden index maximizes difference between TPR (sensitivity) and FPR (1 - specificity): Youden index = TPR - FPR = sensitivity + specificity - 1. Thus, by maximizing “sensitivity + specificity” across various cutoff points, the optimal cutoff point was calculated.

To determine statistical differences for histological, behavioral and molecular expression data, we performed Student's t test (paired and unpaired), and one- and two-way ANOVA (ordinary and repeated measures) using GraphPad Prism 9.0 (GraphPad Software, Boston, USA). Tukey's or Sidak's post hoc analysis was applied, when applicable, to correct for multiple comparisons. Statistical significance was aet as *P < 0.05, **P < 0.01, ***P < 0.0001. All data are presented as means ± SEM. All statistical detail can be found in the Supplementary Data (statistical results).

## Results

### A subpopulation of mice is vulnerable to chronic stress and shows depressive-like behaviors

**Figure 1:**
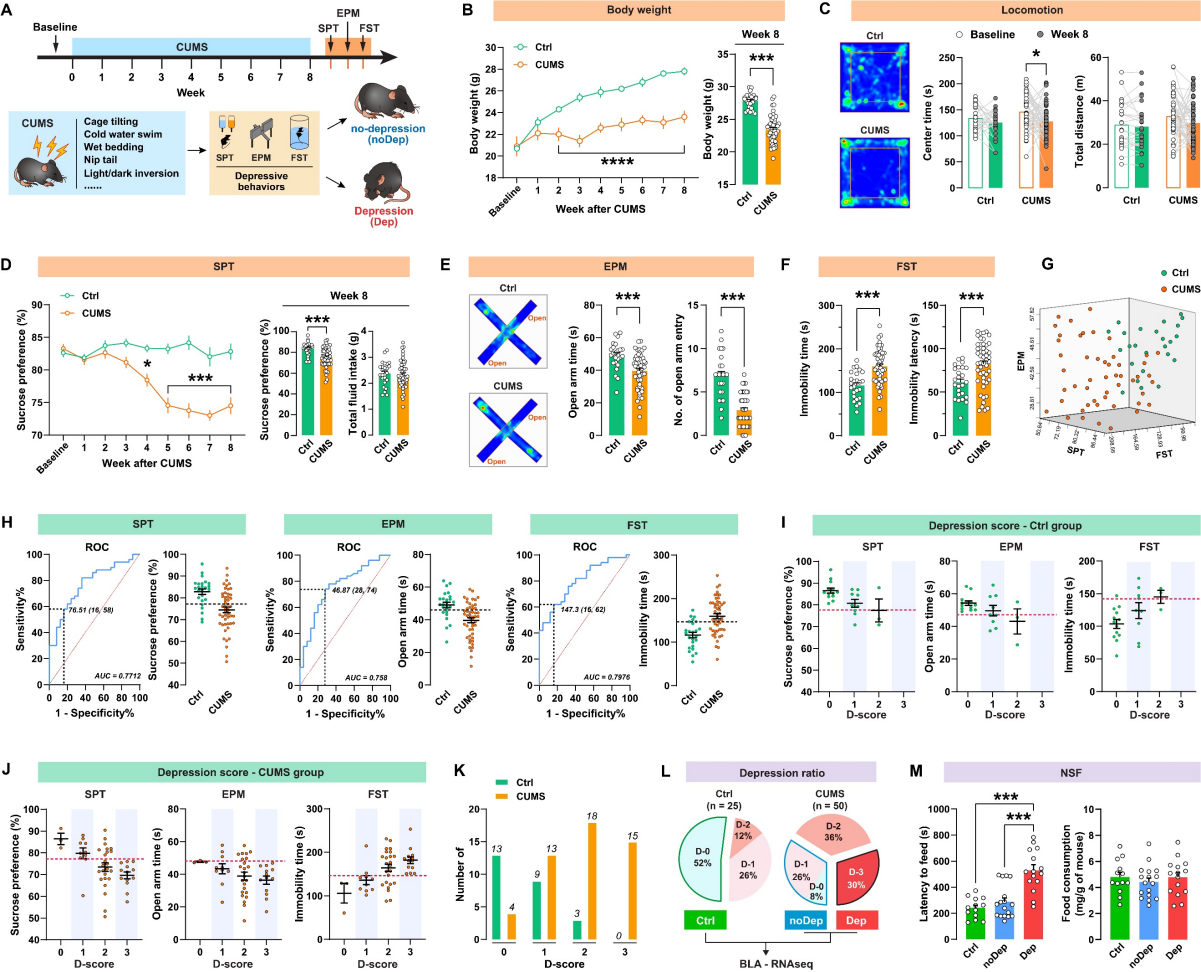
** A subpopulation of mice is vulnerable to chronic stress and shows depressive-like behaviors. A,** Timeline of the CUMS paradigm and behavioral assessments in mice. **B,** Body weight of mice during 8 weeks of CUMS period. **C,** Representative traces in OFT. The total distance traveled and time spent in the OFT center zone were analyzed. **D,** Sucrose preference of mice during the CUMS period. Total fluid intake was also analyzed at week 8. **E,** Representative traces in EPM. Time spent and number of entries in open arms were recorded, respectively. **F,** Immobility time and latency to first immobility in FST. **G,** Three-dimensional plot showing the variability in depressive behavior in SPT, EPM, and FST. **H,** ROC algorithm to establish cutoff criteria based on SPT, EPM, and FST. Dash line represents cutoff value based on the Youden index. AUC, area under curve. Ctrl: n = 25; CUMS: n = 50. **I-J,** Depression scores in Ctrl and CUMS group. Mice were considered positive for behavioral criteria 0 to 3 according to the cutoff value (dash line). **K,** Number of animals distributed among different depression scores. **L,** Separation of groups according to depression score. CUMS mice that met all three positive criteria (D-3) were classified as the depressive (Dep) phenotype. CUMS mice with D-0 or D-1 were classified as the nondepressive (noDep) phenotype. The Ctrl group only included mice with D-0. BLA tissue from the 3 groups of mice was used for transcriptome resequencing. **M,** NSF test was conducted to validate the grouping of mice. Latency to feed and total food consumption were analyzed. Data are presented as means ± SEM. *P < 0.05, ***P < 0.0001, compared to Ctrl or the indicated group.

The timeline of the CUMS paradigm and the behavioral assessments is shown in Fig. 1A. Mice were exposed to 8 weeks of CUMS and subsequently analyzed for depressive-like behaviors. As shown in Fig. 1B, CUMS-exposed mice had significantly decreased body weight compared to non-stressed control (Ctrl) mice from week 2. This decrease in body weight was still significant at 8 weeks. In the open-field test (Fig.1 C), the CUMS mice showed a decrease in center time, while locomotion did not change. At the end of the CUMS, we used SPT to determine whether the mice showed anhedonia, which is a core symptom of major depression 28. CUMS mice exhibited a significant decrease in sucrose preference from week 4 to 8 (Fig. 1D), indicating that this CUMS paradigm is effective and sufficient to induce anhedonia in mice. Anxiety-related behavior and pathological motivational impairments are also commonly seen in patients with major depression. We found that CUMS mice spent significantly less time and had fewer entries in open arms in EPM (Fig. 1E) and struggled significantly less in FST (Fig. 1F), suggesting that these mice developed evident anxiety and despair in response to chronic stress. There were significant correlations between sucrose preference, open-arm time, and immobility time within the same animals (Supplementary Fig. S1), suggesting good coherence of these measures for the assessment of depressive-like phenotypes in mice. We noticed that the effect of the CUMS paradigm in inducing depressive-like behaviors was overall significant, but individual variability was still large (Fig. 1G). Therefore, it was necessary to distinguish subpopulations that are more vulnerable to chronic stress. As described in our previous study 20, we used the receiver operating characteristic (ROC) algorithm to establish cutoff criteria based on the three depressive measures. The area under curve (AUC) for all measures is greater than 0.7, suggesting good diagnostic accuracy for these measures (Fig. 1H). According to the Youden index 29, the cut-off values that have the combination of the highest true positive and lowest false positive rates are 76.51% for SPT sucrose preference, 46.87s for EPM open-arm time, and 147.3s for FST immobility time.

Next, we identified the most vulnerable individuals in the Ctrl and CUMS mice. The screening method used was similar to that described in the study by Cerniauskas *et al.*, but with some modifications 30. According to the number of positive criteria met, a depression score (D-score) between 0 and 3 was assigned to these mice. Both the Ctrl and CUMS groups contained animals that were positive for one to three criteria or did not show depressive-like behaviors (Fig. 1I-J). However, most of the mice in the Ctrl group had low depression scores, while the mice in the CUMS group had higher depression scores (Fig. 1K). More specifically, mice that met all three depressive criteria (i.e. D-score = 3) represented 30% of the CUMS group, while 34% of these mice met none or only one of the three depressive criteria (i.e. D-score = 0 or 1). In contrast, 52% of the Ctrl mice had a D-score of zero (Fig. 1L). Therefore, the depressive mice (Dep) were identified as those that met all three positive criteria (D-score = 3). The non-depressive mice (noDep) were identified as D-score = 0 and 1. The Ctrl group only included mice that did not show any depressive-like phenotype (D-score = 0). This method was used for subsequent experiments to screen out the depressive mice in the CUMS model. We further validated the Dep and noDep subgroups by the novelty suppressed feeding test. Although total food consumption did not differ between the two groups, Dep mice had significantly higher food collection latency than noDep mice, demonstrating more severe depressive-like states in Dep mice (Fig. 1M).

### Hippo pathway is specifically altered in BLA of mice with depressive-like phenotype

**Figure 2:**
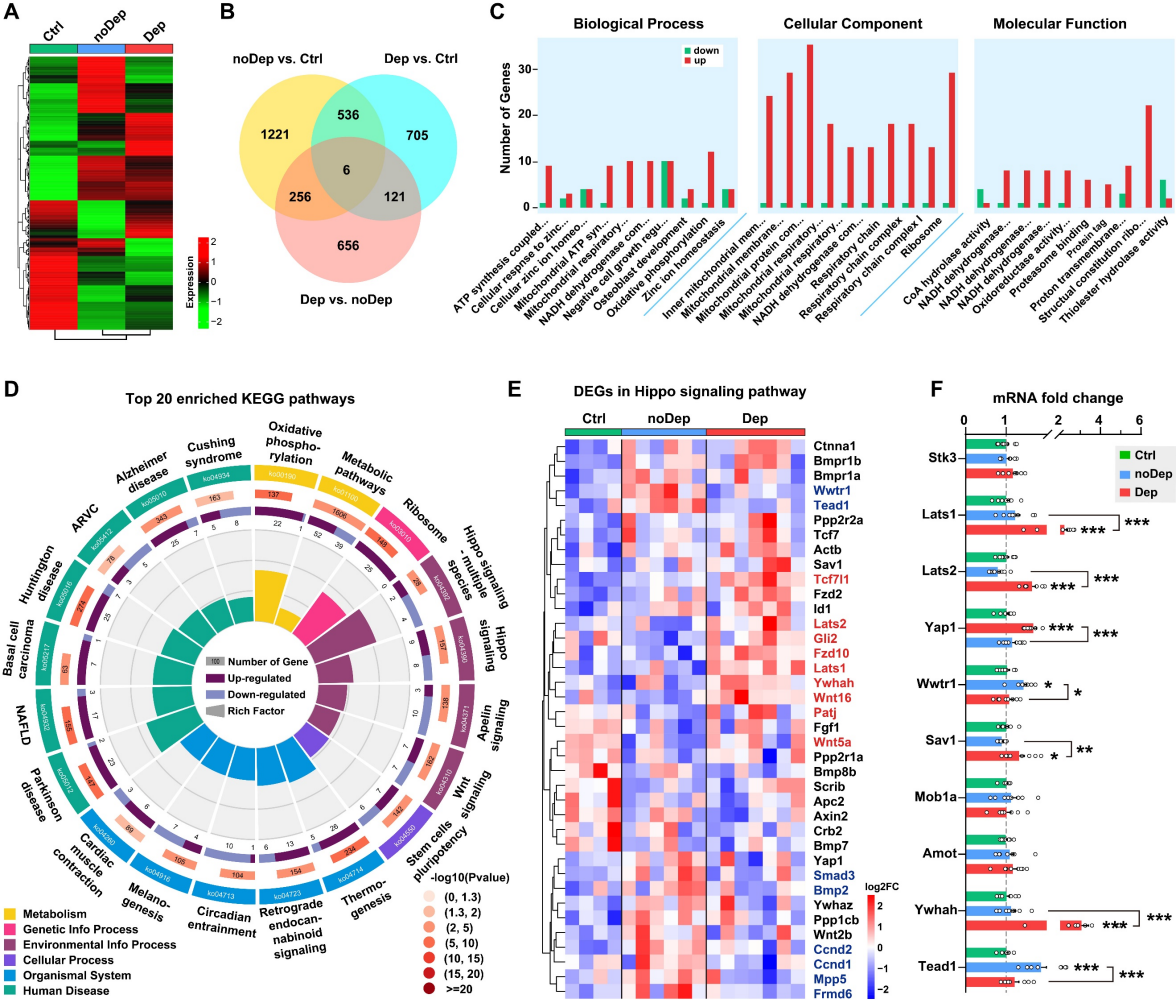
** Transcriptomic alterations and selected Hippo pathways by RNA sequencing in BLA. A**, Heatmap of differentially expressed gene (DEG) sets showing expression trends and hierarchical clusters, with the green to red gradient depicting downregulation to upregulation. Ctrl: n = 4; noDep: n = 6; Dep: n = 7. **B,** Venn diagram of DEGs from different comparison groups. **C,** GO enrichment analysis of DEGs identified in Dep vs. noDep. Top ten GO terms in biological processes, cellular component, and molecular function were shown. **D,** Top 20 enriched KEGG pathways of DEGs identified in Dep vs. noDep. **E,** Heatmap of DEGs in the Hippo pathway from different comparison groups. Names of genes in red or blue color denote upregulated or downregulated DEGs identified in Dep vs. noDep. **F,** mRNA expression levels of key DEGs in the Hippo pathway by qRT-PCR analysis. Data are presented as means ± SEM. *P < 0.05, **P < 0.01, ***P < 0.0001, compared to Ctrl or the indicated group.

We used the RNA sequencing approach to identify specific molecular changes in BLA of mice with nondepressive- or depressive-like phenotype. Of approximately 54,500 transcripts present in the annotation set, a total of 14,234 transcripts were expressed above the detection threshold (10 reads/sample). Heat maps of the expression from the transcriptome data clearly indicated that several clusters of differentially expressed genes (DEGs) were uniquely changed in Dep mice, while the same DEGs in the noDep group were altered in the opposite direction (Fig. 2A and Supplementary Fig. S2A), which emphasizes the dysregulation of gene expression in the BLA of Dep mice. To evaluate the differences in gene expression between the Dep, noDep, and Ctrl groups, a Venn diagram of the overlapping DEGs was plotted (Fig. 2B; see Supplementary Table S3 for the full list of DEGs). In particular, Dep vs. noDep has 1039 DEGs among which 502 were upregulated and 537 were downregulated. Fig. 2C showed the GO enrichment analysis of DEGs identified in Dep vs. noDep. Most of the top biological processes and molecular function identified related to ATP production and mitochondrial metabolism. The identified cellular components were mitochondrial organelle, respiratory chain complex, and cytosolic ribosome. The top three molecular functions include protein binding, catalytic activity, and molecular function regulator. GO enrichment analysis were also performed on Dep vs. Ctrl and noDep vs. Ctrl (Supplementary Table S4). All data suggested unique biological processes, molecular functions, and cellular components in the BLA of Dep mice. Further KEGG enrichment analysis suggested that “Hippo signaling pathway-multiple species”, “Parkinson disease”, “Ribosome”, and “Oxidative phosphorylation” were four most significantly enriched pathways (Fig. 2D; rich factor 0.214 ~ 0.168). It is noteworthy that the “Hippo signaling pathway” (ko04390) was among the most significantly enriched pathways in the KEGG category of “Signal transduction” (rich factor 0.115, *P*adj. = 0.00405). All results of the KEGG pathway analysis are presented in the Supplementary Fig. S2B-D and Supplementary Table S5.

The Hippo pathway is a conserved signaling cascade that regulates mitochondrial function and tissue growth, primarily dealing with cell survival, proliferation, and apoptosis. Recent studies have highlighted the increasing role of the Hippo pathway in brain development, synaptogenesis, and neuronal degeneration 31, 32. Therefore, we aimed to investigate whether this pathway is critically involved in the susceptibility to chronic stress. A total of 38 genes in the Hippo pathway were differentially expressed in Dep vs. Ctrl or noDep vs. Ctrl, while 9 genes were significantly upregulated and 8 genes were downregulated in Dep vs. noDep (Fig. 2E, Supplementary Fig. S2E, Supplementary Table S6). We validated the expression levels of some key genes in the Hippo pathway by qRT-PCR. Lats1, Lats2, Sav1, and Ywhah were significantly upregulated, while Yap1, Wwtr1, and Tead1 were significantly downregulated in Dep vs. noDep (Fig. 2F). These results clearly indicated the dysregulation of the Hippo pathway in BLA of mice with depressive-like phenotype.

### Chronic stress gradually activates the Hippo pathway and inhibits nuclear translocation of YAP in depressive mice

**Figure 3:**
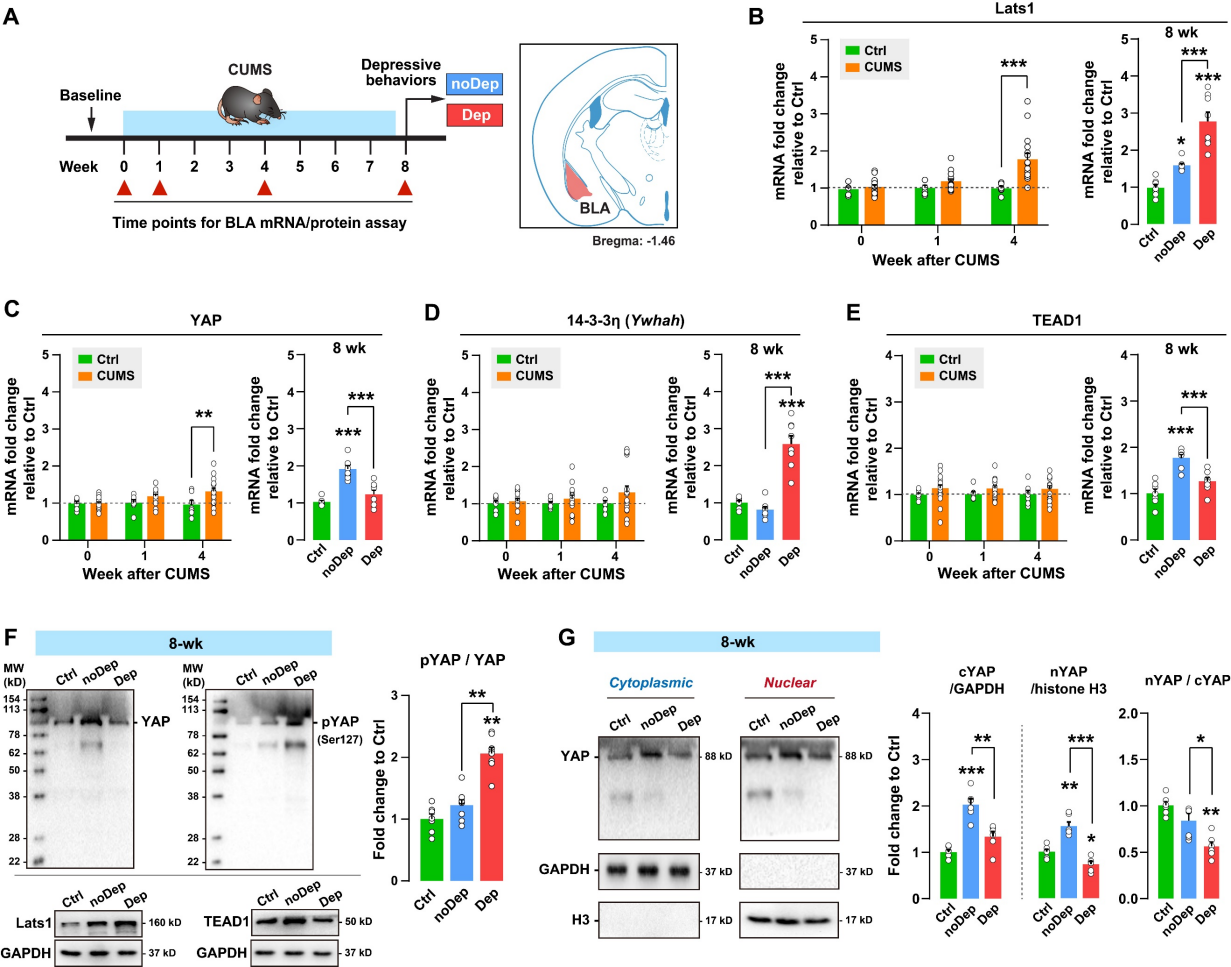
** Alterations in the Hippo pathway and nuclear translocation of YAP in CUMS mice. A,** Timeline of the 8-week CUMS paradigm. BLA samples were collected at different time points for mRNA and protein analysis. **B-E,** mRNA expression level of Lats1, YAP, 14-3-3η (Ywhah), and TEAD1.** F,** Protein expression levels of Lats1, TEAD1, YAP, and phospho-YAP (pYAP, Ser127) in BLA of mice at week 8. The phosphorylation level of YAP was expressed as pYAP to total YAP. Data were normalized to GAPDH. **G,** Expression of YAP in subcellular fractionation. Cytoplasmic and nuclear protein was normalized to GAPDH and histone H3, respectively. Data are presented as means ± SEM. *P < 0.05, **P < 0.01, ***P < 0.0001, compared to Ctrl or the indicated group.

To determine whether dysregulation of the Hippo pathway in BLA varied over the time course of stress, we analyzed the expression of key signaling molecules in CUMS mice over time (Fig. 3A). The expression level of Lats1 mRNA increased significantly in CUMS mice at week 4, while Dep mice showed a higher level of mRNA than noDep mice at week 8 (Fig. 3B). Similarly, CUMS mice showed an increase in the YAP mRNA level at week 4. However, Dep mice showed a significantly lower level of YAP mRNA than noDep mice at week 8 (Fig. 3C). The expression level of 14-3-3η (encoded by *Ywhah* gene) and TEAD1 mRNA did not change during the first 4 weeks of CUMS. However, Dep mice showed a higher 14-3-3η mRNA level and a lower TEAD1 mRNA level than noDep mice at week 8 (Fig. 3D-E). In BLA of 8-week Dep and noDep mice, the abundance of Lats1 and TEAD1 proteins coincided with the change in mRNA expression (Fig. 3F, left). Activation of the Hippo pathway triggers a phosphorylation cascade, which further alters the nuclear localization of YAP and affects target genes 15. In BLA of Dep mice, the abundance of YAP protein was increased in noDep mice but not in Dep mice, in line with the change in mRNA expression. Furthermore, there was a greater increase in Ser127-phospho-YAP (pYAP) in Dep mice, yielding a higher pYAP/YAP ratio, suggesting an inactivation of YAP (Fig. 3F, right). Compared to Ctrl mice, noDep mice showed a significant increase in cytoplasmic-YAP (cYAP, by 2.03-fold), nuclear-YAP (nYAP, by 1.55-fold), while the nYAP/cYAP ratio was relative normal (by 0.82-fold) (Fig. 3G). However, Dep mice had a significantly lower nYAP/cYAP ratio compared to both Ctrl and noDep mice (0.56-fold to Ctrl). These results suggested that chronic stress gradually activated the Hippo pathway and inhibited nuclear translocation of YAP in the BLA of Dep mice.

### Depressive mice showed diverse defectives in synaptic mitochondria in BLA neurons

**Figure 4:**
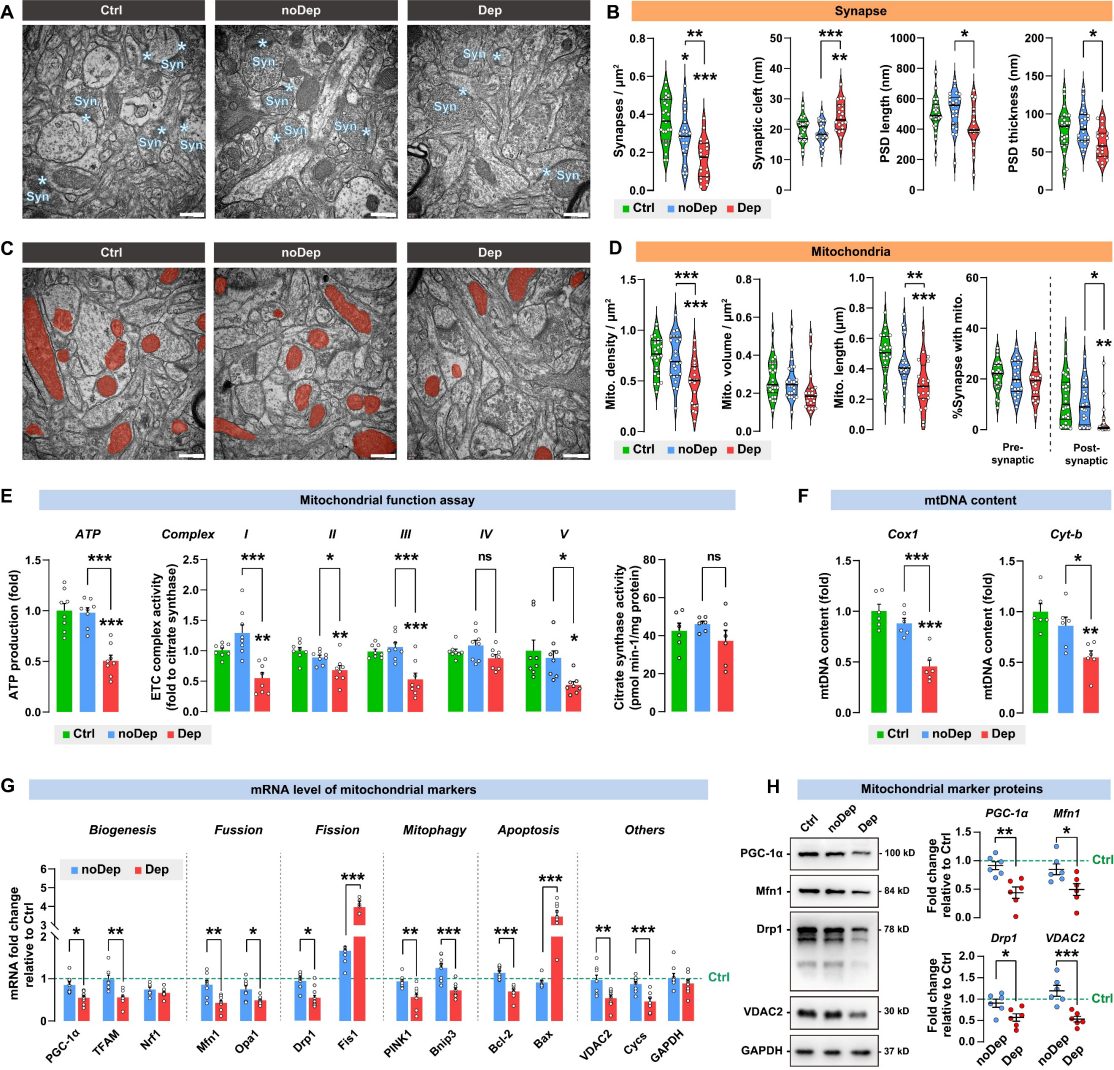
** Ultrastructural abnormalities of synapse and diverse defectives in mitochondria in BLA of depressive mice. A,** Representative EM images of synapses of BLA neurons. Asterisk indicates synapse. Scale bar = 500 nm. **B,** Quantitative measures of synaptic ultrastructure. 4 slices/sample, n = 4-5 mice/group. **C,** Representative EM images of mitochondrion in BLA neurons. Red shade represents mitochondria. Scale bar = 500 nm. **D,** Quantitative measures of the size and density of mitochondrion. 4 slices/ sample, n = 4-5 mice/group. **E-F,** Mitochondrial function was measured by mitochondrial membrane potential, ATP production, ETC complex I activity, and mtDNA content. **G,** mRNA levels of mitochondrial markers in noDep and Dep mice. Data were expressed as fold change relative to Ctrl group (dash line). **H,** Protein levels of mitochondrial makers in noDep and Dep mice. Data were expressed as fold change relative to Ctrl group (dash line). Data are presented as means ± SEM. *P < 0.05, **P < 0.01, ***P < 0.0001, compared to Ctrl or the indicated group.

Transmission electron microscopy images were acquired from the mice BLA neurons (Fig. 4A). The noDep mice showed similar synaptic cleft width, postsynaptic density (PSD) length and thickness compared to Ctrl mice (Fig. 4B). However, compared to noDep mice, Dep mice showed a significant decrease in synapse density, PSD length, and PSD thickness, and an increase in synaptic cleft width, suggesting impaired synaptic structures in BLA of Dep mice. Ultrastructural abnormalities of mitochondria in BLA neurons were also analyzed (Fig. 4C). In noDep mice, no changes in the shape or size of the mitochondria were evident. However, in Dep mice, quantitative analysis showed a decrease in mitochondrial density, while the average volume was normal. Other abnormalities included decreased mitochondrial length and reduced number of postsynaptic mitochondria (Fig. 4D). Furthermore, we analyzed mitochondrial functions in these mice. In noDep mice, mitochondrial metabolic function and mtDNA content were not different with Ctrl mice. However, Dep mice showed significant decreases in ATP production, ETC complex I, II, III and V activity, and a lower mtDNA content of the *Cox1* and *Cyt-b* genes. The activity of citrate synthase, which catalyzes the first reaction of the tricarboxylic acid cycle, was not different between the three groups and served as a control of the ETC activity (Fig. 4E-F). By quantitative RT-PCR of BLA extracts, we determined the expression of selected mitochondrial markers mRNA for biogenesis, turnover, mitophagy, apoptosis, and other crucial molecules (Fig. 4G). Compared to noDep mice, Dep mice showed suppressed mitochondrial biogenesis signaling (PGC-1α and TFAM). Markers involved in mitochondrial fusion (Mfn1 and Opa1) or mitophagy (PINK1 and Bnip3) were significantly decreased, while mitochondrial fission (Drp1 and Fis1) and apoptosis (Bcl-2 and Bax) increased significantly. The abundance of VDAC2 and Cycs was also reduced. By immunoblotting, we found a decrease in the protein expression of PGC-1α, Mfn1, Drp1, and VDAC2 in the BLA of Dep mice compared to noDep mice (Fig. 4H). The changes in mitochondrial proteins were in line with the mRNA results. Collectively, these results validated our observations by RNA sequencing and suggested ultrastructural abnormalities of the synapse and various defectives in mitochondria in BLA of Dep mice.

### *In vitro* knockdown or *in vivo* knockout of YAP leads to mitochondrial dysfunction and recapitulates the depressive-like phenotype in mice

**Figure 5:**
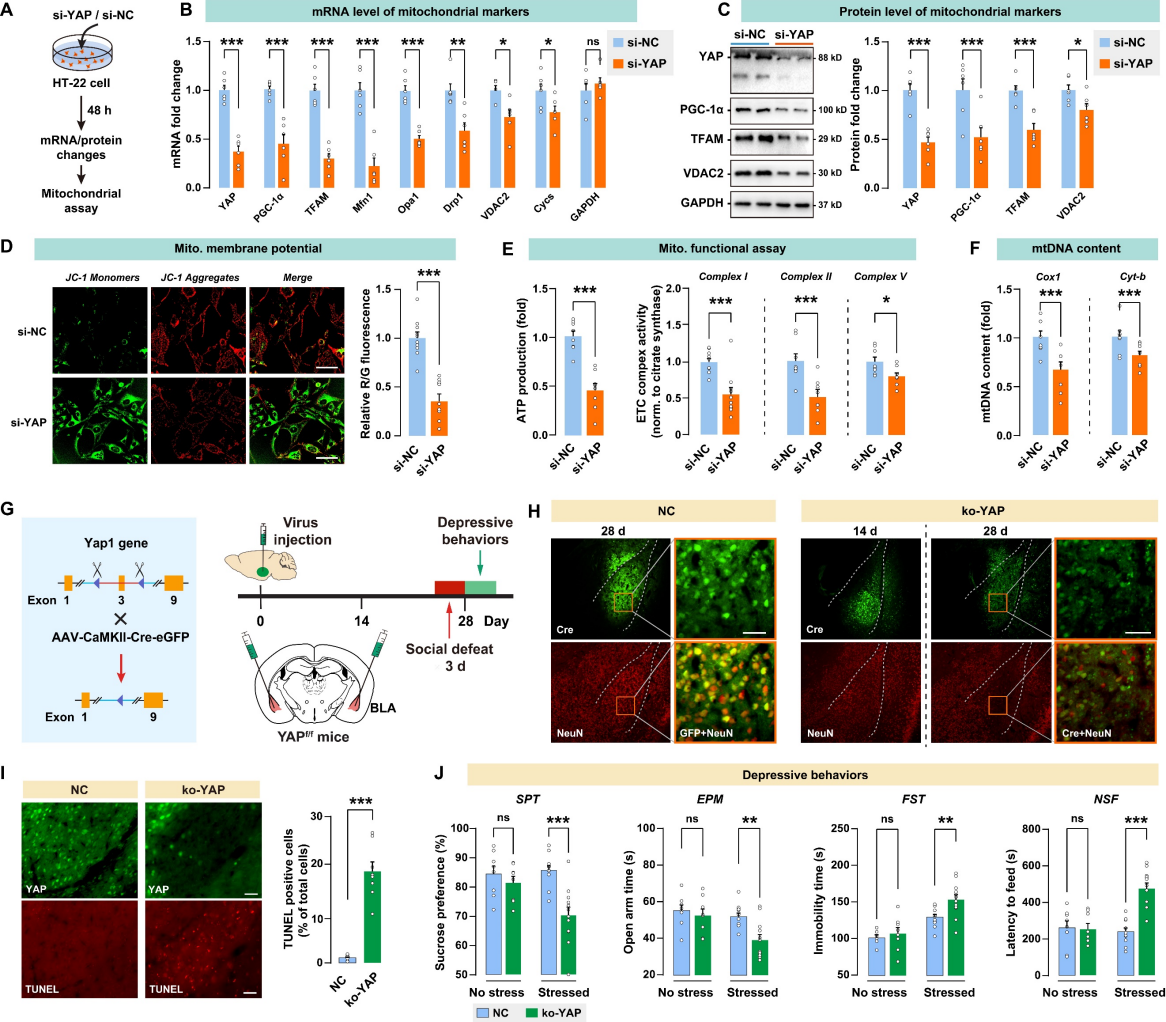
***In vitro* knockdown or *in vivo* knockout of YAP on mitochondrial functions and depressive phenotype in mice. A,** Diagram depicting the workflow of the *in vitro* experiment. HT-22 cells were transfected with siRNA against YAP (si-YAP) or negative control (si-NC). **B,** mRNA levels of mitochondrial markers in si-YAP cells relative to si-NC cells. **C,** Protein levels of mitochondrial markers. **D-F,** Mitochondrial function was measured by mitochondrial membrane potential, ATP production, ETC complex I, II, and V activity, and mtDNA content. Scale bar = 20 μm.** G,** Diagram illustrating the experimental procedure for *in vivo* knockout of YAP in mice. Cre-expressing virus was injected into BLA of floxed Yap1 mice (Yap1^fl/fl^) to achieve conditional knockout of YAP (ko-YAP). n = 8/group. **H,** Representative histofluorescent images of BLA neurons co-labeled with Cre (green) and NeuN (red). Scale bar = 50 μm. **I,** Measurement of apoptosis by TUNEL histofluorescent staining (red) of BLA neurons. Scale bar = 50 μm. **J,** Depressive-like behaviors in mice with or without the 3-day social defeat challenge. Data are presented as means ± SEM. *P < 0.05, **P < 0.01, ***P < 0.0001, compared to the NC group.

To investigate the impact of YAP activity on mitochondrial function, YAP expression was knocked down using siRNA in a mouse neuronal HT-22 cell line (Fig. 5A). A significant decrease in YAP mRNA was observed in the si-YAP group, with the mRNA level reduced by 0.37-fold in HT-22 cells over the 48-h course (Fig. 5B). Furthermore, HT-22 cells treated with YAP siRNA showed significantly reduced mitochondrial marker mRNA levels, such as PGC-1α, TFAM, Mfn1, Opa1, Drp1, VDAC2, and Cycs. Congruently, siRNA suppressed the level of the YAP protein by 0.49-fold, and decreased PGC-1α, TFAM, and VDAC2 proteins in HT-22 cells (Fig. 5C). JC-1 analysis revealed a significant decrease in red/green fluorescence for si-YAP cells compared to negative control (si-NC) cells, indicating that YAP knockdown impaired mitochondrial membrane potential (Fig. 5D). YAP knockdown also significantly reduced the ATP production and ETC complex activity in HT-22 cells (Fig. 5E). Furthermore, YAP knockdown leads to a decrease in mtDNA content, as measured by the mitochondrial encoded gene Cox1 and Cyt-b. These results suggested that the knockdown of YAP *in vitro* leads to mitochondrial dysfunction.

To investigate the *in vivo* effect of YAP activity in BLA on depressive-like behaviors, Yap1 floxed mice (Yap1^fl/fl^) received bilateral injection of AAV-CaMK2-Cre-eGFP into BLA to knockout YAP expression (ko-YAP) in glutamatergic neurons. AAV-CaMK2-eGFP was also injected into BLA as the negative control (NC). Depressive-like behaviors were evaluated 28 days after virus injection. On days 25 to 27, mice were subjected to 3 days of social defeat to precipitate depressive-like behaviors (Fig. 5G). The AAV-mediated YAP knockout was confirmed by immunoblotting of YAP in BLA (Supplementary Fig. S3A). We also validated the specificity of the virus by confirming its interaction with CaMK2 and GAD67 in BLA (Supplementary Fig. S4). After 28 days of virus injection, there was a notable reduction in the number of BLA neurons that co-express both GFP and the neuronal marker NeuN, as shown by immunostaining (Fig. 5H). TUNEL positive cells in the ko-YAP group increased significantly compared to the NC group, confirming that YAP knockout promoted apoptosis in BLA neurons (Fig. 5I). Next, we examined whether YAP knockout in BLA affects stress-induced depressive behaviors in mice. After 28 days of virus injection, spontaneous depressive behaviors were not detected in NC and ko-YAP mice that are not subjected to any stress (Fig. 5J). We found that the subthreshold social defeat stress failed to trigger depressive-like behavior in NC and ko-YAP mice (Supplementary Fig. S5). However, after 3 consecutive days of social defeat stress, ko-YAP mice showed a significant decrease in SPT sucrose preference, EPM open arm time, and an increase in FST immobility time and NSF feeding latency compared to the NC group.

In addition, we found that Yap knockout in BLA GABAergic neurons has no significant effect on depressive-like phenotype, at least on the depressive susceptibility induced by the 3-day social defeat stress (Supplementary Fig. S6). Taken together, these results demonstrated that the knockout of YAP *in vivo* leads to neuronal apoptosis and increases susceptibility to stress-induced depressive-like phenotype in mice.

### Upregulation of YAP in BLA abates stress-induced depressive-like phenotype in mice

**Figure 6:**
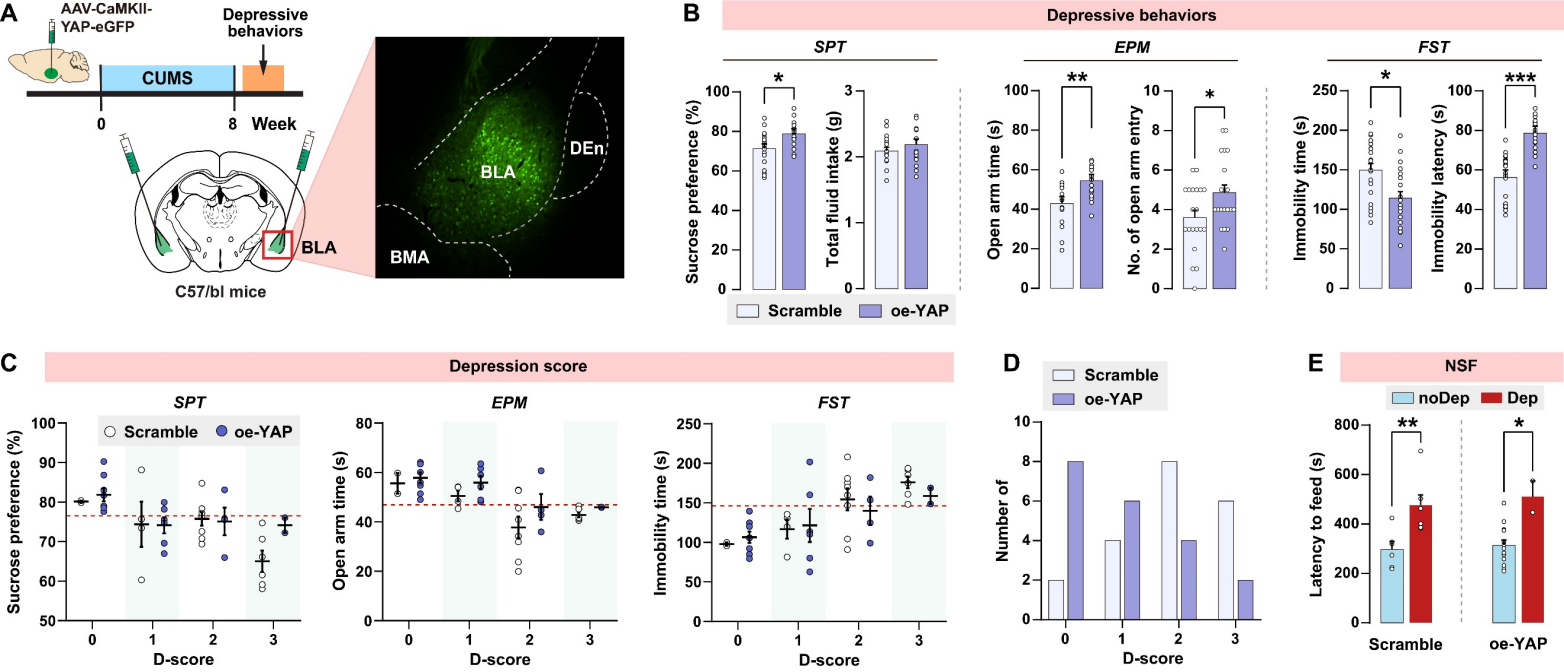
***In vivo* overexpression of YAP on depressive phenotype in mice. A,** Diagram illustrating the experimental procedure for virus-mediated overexpression of YAP (oe-YAP) in BLA of mice. Representative histofluorescent image showing the virus transfection range. n = 20/group. **B,** CUMS-induced depressive-like behaviors in mice of oe-YAP and scramble group. **C,** Depression scores in oe-YAP and scramble group. Dash line represents the cutoff value of each depressive behavior. **D,** Number of animals distributed in different depression scores. **E,** Feeding latency of oe-YAP and scramble mice in NSF test. Data are presented as means ± SEM. *P < 0.05, **P < 0.01, ***P < 0.0001, compared to scramble or the indicated group.

Since *in vivo* knockout of YAP in BLA recapitulates the depressive-like phenotype seen in CUMS mice, we explored whether upregulation of YAP in BLA prevents CUMS-induced depressive-like behaviors in mice. Wild-type C57/BL6J mice were bilaterally injected with YAP-overexpressing virus in BLA glutamatergic neurons prior to the CUMS paradigm (Fig. 6A). AAV-mediated overexpression of YAP (oe-YAP) was confirmed by immunoblotting (Supplementary Fig. S3B). Interestingly, after 8 weeks of CUMS, mice in the oe-YAP group exhibited higher levels of sucrose preference, open arm time and entries, and lower levels of immobility time compared to mice transfected with the scramble control virus (Fig. 6B). In accordance with the depressive criteria defined in the previous experiment, we identified the Dep and noDep mice in both the scramble and oe-YAP groups. Most of the mice in the oe-YAP group had lower depression scores compared to the mice in the scramble control group (Fig. 6C and D). The depressive phenotypes in both the scramble and oe-YAP groups were testified by NSF feeding latency (Fig. 6E). In summary, *in vivo* overexpression experiment showed that upregulation of YAP in BLA increased mice's resilience to stress-induced depressive-like phenotype.

### Integrative Hippo/YAP pathway mediates mitochondrial dysfunction and is essential for stress-induced depressive-like phenotype in mice

**Figure 7:**
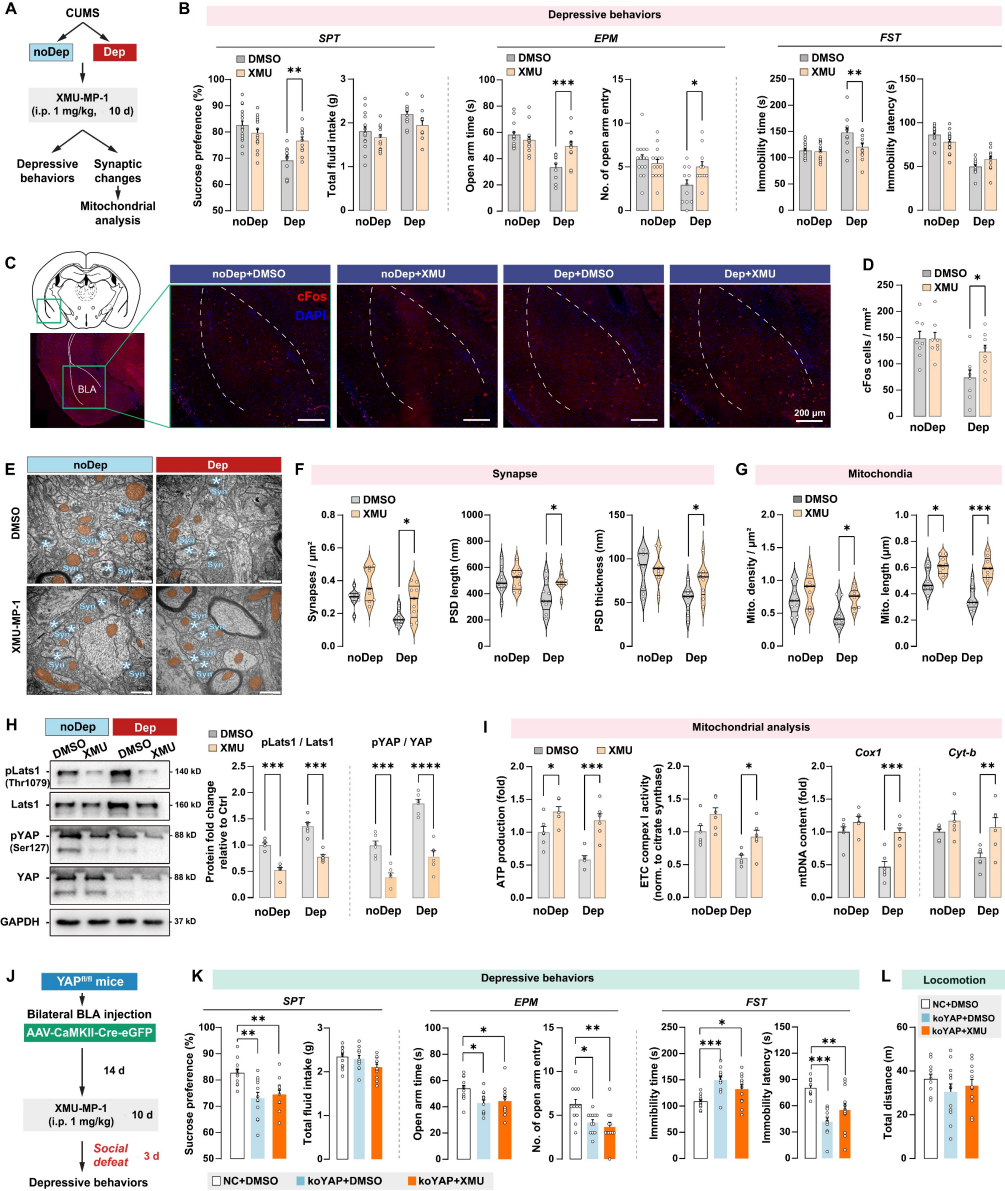
** Hippo/YAP pathway mediates mitochondrial function and depressive phenotype in mice. A,** Schematic diagram of the Mst1/2 inhibition experiment. Mice from the noDep and Dep groups were treated with XMU-MP-1, a selective Mst1/2 inhibitor, and were tested for depressive behaviors. n = 11-15/group. **B,** Depressive behaviors in XMU-MP-1 or DMSO-treated mice. **C,** Representative immunofluorescent images showing the cFos expression in BLA. Scale bar = 200 μm. **D,** Analysis of cFos positive cells in BLA. n = 8 mice/group. **E,** Representative EM images showing the ultrastructure of BLA neurons. Scale bar = 500 nm. **F,** Quantitative measures of synaptic ultrastructure. 6 slices/BLA sample, n = 4 mice/group. **G,** Quantitative measures of the size and density of mitochondria. **H,** Phosphorylation levels of Lats1 and YAP in BLA of mice. **I,** Mitochondria function was measured by mitochondrial membrane potential, ATP production, ETC complex I activity, and mtDNA content. **J,** Schematic diagram of intra-BLA YAP knockout (koYAP) combined with Mst1/2 inhibition in mice. n = 12/group. **K,** Depressive behaviors in different treatment groups. **L,** Locomotion of mice in OFT. Data are presented as means ± SEM. *P < 0.05, **P < 0.01, ***P < 0.0001, compared to DMSO or the indicated group.

Mst1/2 are critical components of the Hippo signaling pathway and core kinases that phosphorylate Lats1/2 kinases, which in turn phosphorylate YAP and inhibit its nuclear translocation 33. Since CUMS induced YAP phosphorylation in BLA of Dep mice, we investigated the effects of pharmacological inhibition of Mst1/2 using a selective inhibitor XMU‐MP‐1 in regulating depressive-like behaviors and mitochondrial dysfunction. After the CUMS paradigm, Dep and noDep mice were injected with XMU‐MP‐1 (i.p, 1 mg/kg) for 10 consecutive days (Fig. 7A). The XMU‐MP‐1 dosing regimen was based on the pharmacokinetics data in rodents 34, 35. Treatment with XMU-MP-1 significantly reversed low sucrose preference and EPM open arm time and entries, and reduced FST immobility time in Dep mice, while it did not affect depressive-like behaviors in noDep mice (Fig. 7B). Using immunostaining, we analyzed cFos expression in BLA in mice (Fig. 7C). There were less cFos+ cells in Dep mice compared to noDep mice, while treatment with XMU-MP-1 significantly increased cFos expression in Dep mice (Fig. 7D). These results indicate that blocking the Hippo signaling pathway may improve BLA activity in Dep mice. Using TEM, we analyzed the ultrastructural changes in BLA neurons (Fig. 7E). In Dep mice, XMU-MP-1 significantly increased synapse density, PSD length, and thickness compared to the DMSO vehicle group (Fig. 7F), indicating an improvement in synaptic abnormality. In noDep mice, no changes in the synapses of BLA neurons were evident. Furthermore, XMU-MP-1 treatment resulted in an increase in both mitochondrial density and length in Dep mice, while noDep mice only exhibited an increase in mitochondrial length (Fig. 7G). Immunoblotting confirmed that XMU-MP-1 treatment strongly repressed the phosphorylation of Lats1/2 and YAP in both Dep and noDep mice (Fig. 7H). In Dep mice treated with XMU-MP-1, we found that mitochondrial ATP production, ETC complex activity, and mtDNA content increased significantly, indicating that pharmacological inhibition of Mst1/2 promoted mitochondrial function of BLA neurons (Fig. 7I).

To verify that an integrative Hippo/YAP pathway in BLA is required for the stress-induced depressive-like phenotype, we jointly applied intra-BLA YAP knockout and systemic Mst1/2 inhibition and detected stress-induced depressive-like behavior in mice (Fig. 7J). As expected, YAP (ko-YAP) knockout in BLA neurons led to a significant stress-induced depressive-like phenotype (Fig. 7K), which is consistent with our observation in the previous YAP knockout experiment. However, the improvement effect of XMU-MP-1 on depressive-like behaviors was not observed in ko-YAP mice. No changes in locomotion were evident in all treatment groups (Fig. 7L). These results suggested that an integrative Hippo/YAP pathway in BLA was essential for the stress-induced depressive-like phenotype in mice.

### 14-3-3η interacts with Hippo/YAP pathway and is involved in stress-induced depressive-like phenotype

**Figure 8:**
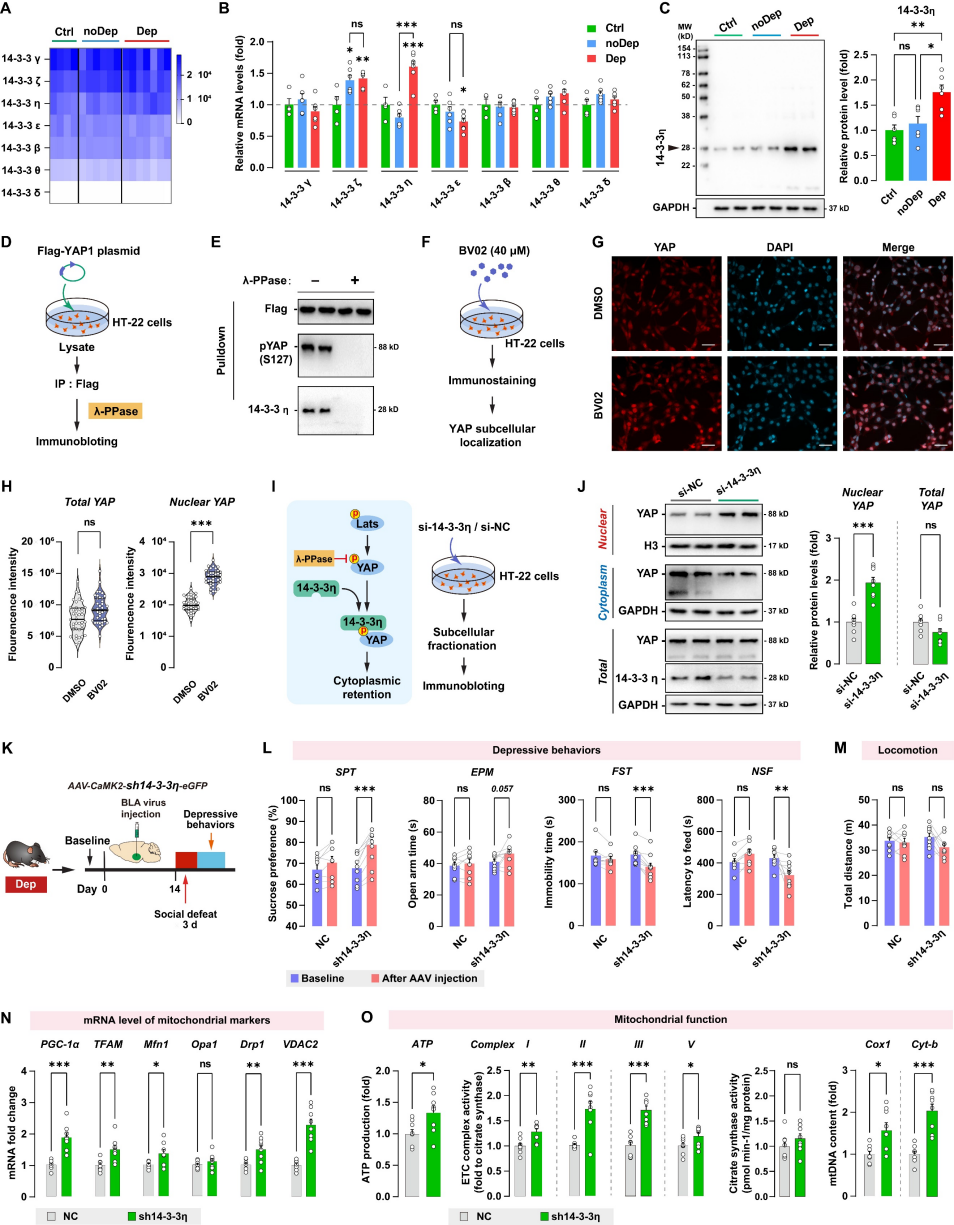
** 14-3-3η/YAP interaction regulates mitochondrial function and depressive phenotype in mice. A,** Heatmap of transcriptome analyses showing mRNA expression levels of 7 isoforms of the 14-3-3 proteins. **B,** mRNA levels of 14-3-3 isoforms in noDep and Dep mice. Data were expressed as fold change relative to Ctrl group (dash line).** C,** Protein levels of 14-3-3η in noDep and Dep mice. Data were normalized to GAPDH. **D,** Diagram depicting the workflow of the in vitro validation for 14-3-3η/YAP interaction. HT-22 cells were transfected with Flag-YAP1 plasmid. Flag-YAP1 was immunoprecipitated and treated with λ phosphatase and then used to pulldown endogenous 14-3-3η from lysate. **E,** Immunoblotting result showing the 14-3-3η/YAP interaction, and this interaction was completely abolished by phosphatase treatment. **F,** Diagram for the in vitro inhibition of 14-3-3 protein. HT-22 cells were treated with BV02, a small-molecule pan-14-3-3 inhibitor. YAP subcellular localization was analyzed by immunofluorescence. **G,** Representative histofluorescent images of DMSO- or BV02-treated HT-22 cells. Scale bar=50 μm. **H,** Quantitative analysis showing increased accumulation of nuclear YAP in BV02-treated HT-22 cells. **I,** *Left*: diagram depicting the proposed mechanism of 14-3-3η regulating YAP cytoplasmic retention. *Right*: diagram for the in vitro 14-3-3η knockdown experiment. HT-22 cells were transfected with siRNA against 14-3-3η. **J,** Expression of YAP in subcellular fractionation. Cytoplasmic, nuclear, and total extracts from HT-22 cells with 14-3-3η knockdown were immunoblotted. Cytoplasmic and nuclear protein was normalized to GAPDH and histone H3, respectively. **K,** Timeline of the intra-BLA knockdown of 14-3-3η and subsequent behavioral assessments in Dep mice. n = 8-9/group. **L,** Depressive behaviors in different treatment groups of mice. **M,** Locomotion of mice in OFT. **N,** mRNA levels of mitochondrial markers in BLA of mice. **O,** Mitochondrial function was measured by mitochondrial membrane potential (JC1 assay), ATP production, ETC complex I, II, and V activity, and mtDNA content. Data are presented as means ± SEM. *P < 0.05, **P < 0.01, ***P < 0.0001, compared to Ctrl, scramble or the indicated group. ns, nonsignificant.

Another important regulatory mechanism of the Hippo/YAP pathway involves its interaction with the chaperone 14-3-3, which is abundantly expressed in the brain. 14-3-3 proteins modulate a wide variety of neuronal signaling processes, and their dysregulation is highly correlated with neurological disorders such as MDD 36 and schizophrenia 37. By analyzing the RNA sequencing data, we found that BLA expressed all seven known isoforms of 14-3-3 proteins, while the γ, ζ, and η isoforms were the most abundantly expressed 14-3-3 proteins (Fig. 8A). The expression of 14-3-3 isoforms in BLA between Ctrl, noDep, and Dep groups was further examined by qPCR (Fig. 8B). Notably, 14-3-3η protein, which is encoded by the *Ywhah* gene, showed a significant increase in the Dep group compared to the Ctrl and noDep group. Increased expression of 14-3-3η protein in BLA of Dep mice was further confirmed by immunoblotting (Fig. 8C). Therefore, we chose to assess specific functions of 14-3-3η isoform and its interactions with the Hippo/YAP pathway. YAP phosphorylation (primarily on Ser127) was reported to be essential to bind to the 14-3-3 protein 38. We transfected the Flag-YAP1 plasmid into HT-22 cells and immunoprecipitated it from HT-22 cell lysate. The precipitate was treated with λ phosphatase and Flag-YAP1 was then used to pulldown endogenous 14-3-3η protein (Fig. 8D). The result confirmed the physical interaction between endogenous YAP and 14-3-3η, and after phosphatase treatment, the binding of 14-3-3η to YAP was completely abolished (Fig. 8E). We further investigated the effect of inhibiting 14-3-3 function on YAP subcellular localization by using immunofluorescence. HT-22 cells were treated with 40 μM BV02, a small-molecule pan-14-3-3 inhibitor 39 (Fig. 8F). While there was no significant change in total YAP intensity, there was a significant increase in nuclear YAP in HT-22 cells treated with BV02 compared to cells treated with DMSO (Fig. 8G and H). Together with these results and previous reports 39, we hypothesized that that 14-3-3η recruits pLats1/2 and YAP to form a complex, which facilitates phosphorylation of YAP and leads to its cytoplasmic retention (Fig. 8I). We used siRNA to knockdown 14-3-3η in HT-22 cells, and then determined the expression of YAP in subcellular fractionation. Knockdown of 14-3-3η significantly increased the nuclear accumulation of YAP, while total YAP levels did not change (Fig. 8J). These results indicated that 14-3-3η interacts with YAP and inhibits the nuclear localization of YAP.

Since 14-3-3η was specifically upregulated in the BLA of Dep mice, we sought to determine whether the *in vivo* knockdown of 14-3-3η in BLA affects stress-induced depressive-like behaviors. Dep mice were exposed to a 3-day social defeat paradigm 14 days after receiving bilateral BLA virus injection (expressing shRNA against 14-3-3η) and retested for depressive-like behaviors (Fig. 8K). The efficiency of shRNA-induced *in vivo* knockdown of the YAP protein was verified by immunoblotting (Supplementary Fig. S3C). Knockdown of 14-3-3η in BLA produced a higher level of sucrose preference and a lower level of immobility time and feeding latency compared to mice behavior before virus injection (Fig. 8L). Furthermore, 14-3-3η knockdown in BLA produced a trend towards an increase (*P* = 0.057) in open arm time in Dep mice. No changes in depressive-like behaviors were found in mice transfected with the NC control virus. No changes in locomotion were evident in all groups (Fig. 8M). By qRT-PCR analysis of BLA samples, we found that mRNA levels of PGC-1α, TFAM, Mfn1, Drp1, and VDAC2 increased significantly in the sh14-3-3η group (Fig. 8N). Furthermore, mitochondrial ATP production, ETC complex activity, and mtDNA content also increased in the sh14-3-3η group, indicated that the knockdown of 14-3-3η enhanced mitochondrial function in BLA neurons (Fig. 8O). In summary, *in vivo* knockdown experiment showed that downregulation of 14-3-3η in BLA improved mitochondrial dysfunction and stress-induced depressive-like phenotype in mice.

## Discussion

The current study presents several novel findings on the mouse model of CUMS-induced depressive behaviors. We provided a behavioral screening method based on the ROC algorithm. This method allowed us to identify a specific subpopulation of mice that were exposed to CUMS and exhibited the most prominent depressive phenotype. From the transcriptomic data of these mice, we identified a large number of genes and signaling pathways that were specifically altered from the BLA of depressive (Dep) mice, relative to the nondepressive (noDep) or control (Ctrl) mice.

From a cellular perspective, Dep mice displayed notable synaptic impairment in BLA neurons, as well as mitochondrial damage characterized by abnormal mitochondrial morphology, compromised function, impaired biogenesis, and alterations in mitochondrial marker proteins. Mechanically, we found that the Hippo signaling pathway was activated in Dep mice during CUMS, and the transcriptional regulatory activity of YAP was suppressed by phosphorylation of its Ser127 site. We also identified 14-3-3η as an important co-regulatory factor of the Hippo/YAP pathway, as it can respond to chronic stress and regulate cytoplasmic retention of YAP. Importantly, the integrated Hippo/YAP/14-3-3η pathway mediated neuronal mitochondrial dysfunction and depressive behavior in Dep mice, suggesting a causal role of this pathway in susceptibility to chronic stress-induced depression.

To begin with, we used a reliable behavioral paradigm to identify mice that exhibit the most significant depressive phenotype after chronic exposure to stress. MDD is a complicated mood disorder with high individual heterogeneity. Despite the diverse pathophysiologies underlying the clinical manifestations of depression, individuals with symptomatic differences are often grouped together in the same diagnostic category 40. Similarly, rodents also exhibit great heterogeneity in responses to stress. However, to our knowledge, few investigators have explicitly examined the individual heterogeneity of depressive phenotypes in rodents in responding to CUMS. In the present study, we directly retrieved metrics from three well-established paradigms of depressive behaviors of CUMS-exposed mice. These behavioral paradigms are well recognized for evaluating depressive behaviors in rodents, including anhedonia (measured by sucrose preference in SPT), anxiety (measured by open arms time in EPM), and behavioral despair (measured by immobility time in FST) 41. Additionally, we incorporated supplementary measures of the depressive state, such as novelty suppressed feeding, weight gain/loss, and locomotion, to enhance the validation of subgroups. The methods for ROC analysis and cut-off value definition based on behavioral metrics are similar to that we previously described 20, 22. We found that 30% of CUMS mice met all three criteria, while 34% of CUMS mice met only one criterion or no criterion. Therefore, we classified CUMS-exposed mice into Dep and noDep subgroups. This method effectively reduced the heterogeneity among mice and improved the specificity for the investigation of underlying neurobiological mechanisms. It is worth noting that several studies have suggested that a single day of subthreshold social defeat stress, combined with manipulation of specific stress-related pathways, could induce depressive-like behaviors 42, 43. These studies mostly used optogenetics and chemogenetics to rapidly and robustly activate or inhibit neurons. We showed that 3 days of social defeat stress rather than the 1-day subthreshold social defeat stress induced significant depressive behaviors in ko-YAP mice, demonstrating that the knockout of YAP leads to susceptibility to stress-induced depressive-like phenotypes. The differences in manipulations, stress paradigms, and molecular pathways may explain the differences from previous studies.

Mitochondria are crucial organelles responsible for ATP production, intracellular Ca^2+^ signaling to establish membrane stability, maintain the balance of reactive oxygen species (ROS) and regulate intricate processes of apoptosis 44. In the brain, mitochondria play a crucial role in neurotransmission, synaptic plasticity, neurogenesis, and neuroinflammation. Several lines of evidence revealed that chronic stress hampers mitochondrial oxidative phosphorylation, dissipates mitochondrial membrane potential, and damages mitochondrial ultrastructure in different brain regions, such as the cortex, hippocampus, and hypothalamus 45, 46. In our hands, there were significant structural abnormalities in the synapses of BLA neurons from Dep mice, but not in noDep or Ctrl mice. These changes include a decrease in synaptic density, an increase in synaptic cleft, and a shortened and thin PSD, which indicated an impairment in neuronal plasticity in BLA of Dep mice. The structural and functional abnormalities of the mitochondria were also evident in Dep mice. Abnormal mitochondrial turnover was strongly indicated by low levels of marker proteins for biogenesis (e.g., PGC-1α, TFAM), fusion (e.g., Mfn, Opa, Fis), and mitophagy (PINK1), a crucial process in mitochondrial quality control and renewal 47, while fission proteins (e.g., Fis1) were increased. Changes in the apoptosis marker proteins Bax and anti-apoptotic marker Bcl-2 were also evident in Dep mice. In line with our results, studies found that the activation of the Bax and Bcl-2 antagonist/killer (BAK) in mitochondria abates cell membrane permeability, ultimately leading to neuronal apoptosis 48. Therefore, our findings strongly suggest that chronic stress-induced depressive behaviors may be attributed to energy impairment in the BLA caused by mitochondrial dysfunction.

Transcriptome data suggest that a larger number of genes were aberrantly expressed in the BLA of Dep mice compared to noDep or Ctrl mice. In particular, most of the top dysregulated gene clusters are involved in mitochondrial biogenesis, turnover, and ATP production. Integrated pathway analysis identified unique transcriptional disarray in the Hippo signaling pathway in Dep mice. The transcriptome findings were further verified by qRT-PCR and immunoblotting, and the results showed a high degree of consistency. By distinguishing the depressive subpopulations of CUMS-treated mice, we found several intriguing characteristic changes in the Hippo pathway. First, the Hippo pathway was gradually activated by chronic stress, which was particularly significant after 4 weeks of CUMS. The Hippo pathway regulates cell proliferation and apoptosis under diverse extracellular and intracellular stimuli through conserved signaling cascades. Canonical activation of this pathway is well documented in cancer and metabolic and neurodegenerative diseases 49, 50. Recent studies suggest that the Hippo pathway is involved in the pathophysiological processes of stress-related psychiatric disorders. For example, genetic studies have shown an association between gene polymorphisms in certain members of the Hippo pathway (e.g., KIBRA and CLSTN2) and an increased risk of MDD 51. Many receptors that show a strong link with stress and related pathologies (e.g., serotonin 5-HT4, adrenergic α1B, metabotropic glutamate mGlu2) can affect Hippo pathway by activating Rho GTPases 52. Our results provide evidence that the activation of the Hippo pathway in BLA is different between the Dep and noDep subpopulations, suggesting that this pathway may play an important role in the regulation of stress-related signaling. Furthermore, we found that the expression of some key molecules in the Hippo pathway, such as Lats1/2, YAP, TEAD1, was significantly different between the Dep and noDep mice. The main signal output of the Hippo pathway is through YAP. By interacting with transcription factors such as TEAD1, YAP acts as a transcriptional coactivator for numerous genes. Recent studies have shown that loss of YAP expression or inactivation of the YAP/TEAD1 complex induces a perturbation of mitochondrial ultrastructure and function in cells, resulting in activation of apoptosis or necroptosis 53, 54. In line with these reports, we showed a lower nuclear YAP together with transcriptional dysregulation of mitochondrial genes and profound mitochondrial dysfunction in BLA of Dep mice. The crucial role of YAP inactivation in mediating mitochondrial damage was confirmed by our finding, in HT-22 cells, of downregulated mitochondrial markers and following the YAP gene knockdown. More importantly, our findings from the YAP-cKO and overexpression model showed that the YAP signal is critical in mediating the susceptibility of mice depressive behaviors in response to chronic stress. We also found that cFos+ cells were significantly less in Dep mice compared to noDep mice, while XMU-MP-1 treatment significantly increased cFos expression in these mice, suggesting that blocking the Hippo signaling pathway may improve BLA activity in Dep mice. It is worth noting that previous studies have demonstrated hyperactivity of the amygdala during depressive states 55, however, this is not always the case 56-58. Consistent with our findings, Ma *et al.* found that the activity of the posterior BLA to vCA1 innervation was markedly reduced in CUMS mice, while stimulation of this afferent could efficiently alleviate depressive-like behaviors in mice 59. These seemingly contradictory pathological changes are thought to be due to the large differences in experimental design, modeling and the anatomical and functional heterogeneity of BLA along its anterior-posterior axis 59.

The activated Hippo pathway promotes phosphorylation of YAP at different sites through upstream kinases, such as Mst1/2 and Lats1/2, thereby inhibiting the nuclear localization and transcriptional activity of YAP 16. Compared to the noDep group, the expression levels of some key downstream kinases of the Hippo pathway (such as Lat1, Lats2, and Sav1) were significantly higher in the Dep group, and the phosphorylation level of the Ser127 site of YAP showed a corresponding increase. Does Hippo activation/YAP inactivation directly cause mitochondrial damage and is it essential for the stress-induced depressive phenotype of mice? We addressed this question by pharmacological inhibition of Mst1/2 with intra-BLA YAP-cKO and detected stress-induced depressive behaviors in mice. The result indicated that inhibition of the Hippo pathway partially reversed mitochondrial damage and improved depressive behaviors in Dep mice, while YAP is the essential effector for the Hippo pathway to exert these effects. Another interesting finding of this study is that a chaperone protein 14-3-3η (product of the* Ywhah* gene) interacts with YAP and acts as an important co-regulatory factor of the Hippo/YAP pathway. First, 14-3-3η is among the most abundantly expressed 14-3-3 members in BLA, and its expression level in the Dep group was significantly higher than in the noDep group. Through *in vitro* experiments, we further confirmed the interaction between 14-3-3η and the Ser127 phosphorylation site of YAP. This interaction leads to the cytoplasmic retention of YAP and inhibits its transcriptional activity on mitochondrial-related genes. This finding is particularly intriguing because in our previous study we found that the presence of 14-3-3ε in the ventrolateral orbital cortex of mice has a negative regulatory effect on stress-induced mitochondrial damage and neuronal apoptosis 20. The seven isoforms of the 14-3-3 protein have been found to have distinct expression patterns and cellular functions in different cells and tissues 37. In the central nervous system, diverse regulatory functions of 14-3-3 proteins (e.g., θ, ε, γ, and ζ) in neurodegenerative and psychiatric diseases have been reported 60-63. Together, these evidences suggest a complicated role for different isoforms of 14-3-3 proteins in MDD, due to their high heterogeneity in the central nervous system, and deserve more attention and in-depth research.

Our research has several novelties compared to previous studies. Recent studies have shown that the Hippo signaling pathway plays an important role in the nervous system, including neuroinflammation, neuronal differentiation, and neuronal death 64. However, in chronic stress-induced depression, the role of the Hippo signaling pathway and its regulatory effect on mitochondria remains undefined. Duan *et al.* have reported that chronic social defeat caused mitochondrial impairments in the amygdala, which led to activation of the mitophagy pathway 65. However, they focused on the anxiety-like behavior of mice and did not report changes in the depressive phenotype. In molecular mechanisms, a previous study showed that β-adrenoreceptor-Hippo pathway is activated in the Takotsubo syndrome mouse model and mediates mitochondrial damage through inactivation of YAP-TEAD1 in cardiomyocytes 66. Our study further revealed that the Hippo/YAP/14-3-3η signaling pathway regulates mitochondrial function and induces impairments in neuronal synaptic plasticity, contributing to the depressive-like phenotype in mice. Depression is currently recognized as a highly complex brain disease that involves the dysregulation of multiple neural molecular mechanisms. Evidence suggests that the Hippo signaling pathway interacts with various molecular mechanisms, such as the glucocorticoid axis, the Wnt pathway, KIBRA gene mutations, epigenetic regulation, etc., and is involved in stress-related diseases 67. Our research using the RNA sequencing approach not only identified changes in the Hippo/YAP/14-3-3η pathway, but also provided evidence of gene sets of other potential mechanisms for depression.

## Conclusion

We documented notable synaptic impairment and mitochondrial damage in BLA neurons in a subpopulation of mice with the most prominent depressive phenotype. We showed that the Hippo pathway was activated by chronic stress and that the transcriptional regulatory activity of YAP was suppressed by phosphorylation of the Ser127 site. Importantly, the integrated Hippo/YAP/14-3-3η signal is critical in mediating the susceptibility of depressive behaviors in mice in response to chronic stress, suggesting a causal role for this pathway in stress-induced depression. Thus, the use of genetic or pharmaceutical approaches to inhibit the Hippo/YAP/14-3-3η pathway to improve mitochondrial function and ameliorate synaptic impairment in the BLA neuron may provide another avenue for therapeutic development in MDD.

## Supplementary Material

Supplementary data, figures and tables.

## Abbreviations

MDD: major depressive disorder
CUMS: chronic unpredictable mild stress
SPT: sucrose preference test
EPM: elevated plus-maze
FST: forced swim test
OFT: open-field test
Ctrl: control group
ROC: receiver operating characteristic
AUC: area under curve
NSF: novelty suppressed feeding
DEG: differentially expressed gene
EM: electron microscopy
ETC: electron transport chain
AAV: adeno-associated virus
