# Supplementary material for: Chronic stress dysregulates the Hippo/YAP/14-3-3η pathway and induces mitochondrial damage in basolateral amygdala in a mouse model of depression
Source: Theranostics. 2024 Jun 11;14(9):3653–73. doi: 10.7150/thno.92676 (PMC11209716; doi:10.7150/thno.92676)
Supplement: Supplementary file 1 — Supplementary data, figures and tables. [file thnov14p3653s1.zip › Supplementary Data_Statistical results.pdf]

Supplementary Data: Statistical Results

Figure	Measure	Analysis	Effect / Comparison	Test details	P value	Significance
1B	*Left* : Body weight	Two-way RM ANOVA	Interaction	F (8, 584) = 14.86	<0.0001	***
			Time	F (8, 584) = 54.05	<0.0001	***
			CUMS	F (1, 73) = 547.2	<0.0001	***
		Šídák's multiple comparisons test	Baseline	t (657) = 0.3409	>0.9999	ns
		Ctrl vs. CUMS	1wk	t (657) = 2.577	0.0879	ns
			2wk	t (657) = 6.574	<0.0001	***
			3wk	t (657) = 8.424	<0.0001	***
			4wk	t (657) = 8.967	<0.0001	***
			5wk	t (657) = 9.475	<0.0001	***
			6wk	t (657) = 10.12	<0.0001	***
			7wk	t (657) = 11.92	<0.0001	***
			8wk	t (657) = 10.65	<0.0001	***
	Right : Body weight - Week 8	Unpaired t test	Ctrl vs. CUMS	t (73) = 9.723	<0.0001	***
1C	*Left* : OFT - center time	Two-way RM ANOVA	Interaction	F (1, 73) = 0.7518	0.3887	ns
			CUMS	F (1, 73) = 2.120	0.1497	ns
			Time	F (1, 73) = 5.730	0.0192	*
		Šídák's multiple comparisons test	Ctrl	t (73) = 0.9349	0.5813	ns
		Baseline vs. 8 wks	CUMS	t (73) = 2.824	0.0122	*
	Right : OFT - total distance	Two-way RM ANOVA	Interaction	F (1, 73) = 0.6368	0.4275	ns
			CUMS	F (1, 73) = 1.850	0.1779	ns
			Time	F (1, 73) = 1.615	0.2079	ns
1D	*Left* : SPT - Sucrose preference	Two-way RM ANOVA	Interaction	F (8, 584) = 7.751	<0.0001	***
			Time	F (8, 584) = 7.894	<0.0001	***
			CUMS	F (1, 73) = 71.87	<0.0001	***
		Šídák's multiple comparisons test	Baseline	t (657) = 0.4321	>0.9999	ns
		Ctrl vs. CUMS	1wk	t (657) = 0.1726	>0.9999	ns
			2wk	t (657) = 0.7006	0.9974	ns
			3wk	t (657) = 1.926	0.3964	ns
			4wk	t (657) = 3.138	0.0159	*
			5wk	t (657) = 5.594	<0.0001	***
			6wk	t (657) = 6.705	<0.0001	***
			7wk	t (657) = 5.809	<0.0001	***
			8wk	t (657) = 5.357	<0.0001	***
	Right : SPT - Sucrose preference - Week8	Unpaired t test	Ctrl vs. CUMS	t (73) = 4.056	<0.0001	***
	SPT - Total fluid intake - Week8			t (73) = 0.4406	0.6608	ns
1E	*Left* : EPM - Open arm time	Unpaired t test	Ctrl vs. CUMS	t (73) = 0.8807	0.0003	***
	Right : EPM - Open arm entries			t (73) = 7.629	<0.0001	***
1F	*Left* : FST - Immobility time	Unpaired t test	Ctrl vs. CUMS	t (73) = 4.656	<0.0001	***
	Right : EPM - Immobility latency			t (73) = 4.656	0.0007	***
1H	SPT - ROC	Area under the ROC curve		Area = 0.7712	<0.0001	***
	EPM - ROC			Area = 0.7976	<0.0001	***
	FST - ROC			Area = 0.7580	0.0003	**
1M	*Left* : NSF - Latency to feed	One-way ANOVA		F (2, 42) = 21.25	<0.0001	***
		Šídák's multiple comparisons test	Dep vs. Ctrl	t (42) = 5.908	<0.0001	***
			Dep vs. noDep	t (42) = 5.326	<0.0001	***
	Right : NSF - Food consumption	One-way ANOVA		F (2, 42) = 0.4235	0.6575	ns
2F	mRNA fold change Stk3	One-way ANOVA		F (2, 21) = 1.929	0.1702	ns
	Lats1	One-way ANOVA		F (2, 21) = 28.09	<0.0001	***
		Tukey's multiple comparisons test	Ctrl vs. noDep	t (21) = 1.794	0.4278	ns
			Ctrl vs. Dep	t (21) = 9.944	<0.0001	***
			noDep vs. Dep	t (21) = 8.150	<0.0001	***
	Lats2	One-way ANOVA		F (2, 21) = 39.03	<0.0001	***
		Tukey's multiple comparisons test	Ctrl vs. noDep	t (21) = 2.925	0.1209	ns
			Ctrl vs. Dep	t (21) = 9.058	<0.0001	***
			noDep vs. Dep	t (21) = 11.98	<0.0001	***
	Yap1	One-way ANOVA		F (2, 21) = 36.66	<0.0001	***
		Tukey's multiple comparisons test	Ctrl vs. noDep	t (21) = 9.112	<0.0001	***
			Ctrl vs. Dep	t (21) = 2.351	0.2428	ns
			noDep vs. Dep	t (21) = 11.46	<0.0001	***
	Wwtr1	One-way ANOVA		F (2, 21) = 6.808	0.0053	**
		Tukey's multiple comparisons test	Ctrl vs. noDep	t (21) = 4.567	0.0107	*
			Ctrl vs. Dep	t (21) = 0.09777	0.9974	ns
			noDep vs. Dep	t (21) = 4.469	0.0126	*
	Sav1	One-way ANOVA		F (2, 21) = 7.305	0.0039	**
		Tukey's multiple comparisons test	Ctrl vs. noDep	t (21) = 1.339	0.6176	ns
			Ctrl vs. Dep	t (21) = 3.866	0.0320	*
			noDep vs. Dep	t (21) = 5.205	0.0038	**
	Mob1a	One-way ANOVA		F (2, 21) = 0.4649	0.6345	ns
	Amot	One-way ANOVA		F (2, 21) = 0.9534	0.4015	ns
	Ywhah	One-way ANOVA		F (2, 21) = 29.30	<0.0001	***
		Tukey's multiple comparisons test	Ctrl vs. noDep	t (21) = 0.5494	0.9205	ns
			Ctrl vs. Dep	t (21) = 9.639	<0.0001	***
			noDep vs. Dep	t (21) = 9.089	<0.0001	***
	Tead1	One-way ANOVA		F (2, 21) = 20.08	<0.0001	***
		Tukey's multiple comparisons test	Ctrl vs. noDep	t (21) = 8.58	<0.0001	***
			Ctrl vs. Dep	t (21) = 2.049	0.3351	ns
			noDep vs. Dep	t (21) = 6.532	<0.0001	***
3B	*Left* : Lats1 - mRNA fold change	Unpaired t test	0 wk	t (20) = 0.4019	0.9700	ns
		Ctrl vs. CUMS	1 wk	t (20) = 1.063	0.6454	ns
			4 wk	t (20) = 4.512	<0.0001	***
	Right : Lats1 - mRNA fold change - 8 wk	One-way ANOVA		F (2, 20) = 31.90	<0.0001	***
		Tukey's multiple comparisons test	Ctrl vs. noDep	t (20) = 3.711	0.0413	*
			Ctrl vs. Dep	t (20) = 11	<0.0001	***
			noDep vs. Dep	t (20) = 7.55	<0.0001	***
3C	*Left* : YAP - mRNA fold change	Unpaired t test	0 wk	t (20) = 0.0216	>0.9999	ns
		Ctrl vs. CUMS	1 wk	t (20) = 1.528	0.3449	ns
			4 wk	t (20) = 3.178	0.0069	**
	Right : YAP - mRNA fold change - 8 wk	One-way ANOVA		F (2, 20) = 26.68	<0.0001	***
		Tukey's multiple comparisons test	Ctrl vs. noDep	t (20) = 9.726	<0.0001	***
			Ctrl vs. Dep	t (20) = 2.241	0.2752	ns
			noDep vs. Dep	t (20) = 7.748	<0.0001	***
3D	*Left* : 14-3-3h - mRNA fold change	Unpaired t test	0 wk	t (20) = 0.273	0.9902	ns

		Ctrl vs. CUMS	1 wk	t (20) = 0.6287	0.8974	ns
			4 wk	t (20) = 1.516	0.3519	ns
	Right : 14-3-3h - mRNA fold change - 8 wk	One-way ANOVA		F (2, 20) = 46.17	<0.0001	***
		Tukey's multiple comparisons test	Ctrl vs. noDep	t (20) = 1.277	0.6447	ns
			Ctrl vs. Dep	t (20) = 10.76	<0.0001	***
			noDep vs. Dep	t (20) = 12.46	<0.0001	***
3E	*Left* : TEAD1 - mRNA fold change	Unpaired t test	0 wk	t (20) = 1.299	0.4852	ns
		Ctrl vs. CUMS	1 wk	t (20) = 1.15	0.5858	ns
			4 wk	t (20) = 1.074	0.6375	ns
	Right : TEAD1 - mRNA fold change - 8 wk	One-way ANOVA		F (2, 20) = 23.96	<0.0001	***
		Tukey's multiple comparisons test	Ctrl vs. noDep	t (20) = 6.747	<0.0001	***
			Ctrl vs. Dep	t (20) = 2.288	0.0963	ns
			noDep vs. Dep	t (20) = 4.616	<0.0001	***
3F	pYAP/YAP protein fold change	One-way ANOVA		F (2, 20) = 12.53	0.0003	**
		Tukey's multiple comparisons test	Ctrl vs. noDep	t (20) = 0.9564	0.7798	ns
			Ctrl vs. Dep	t (20) = 5.425	0.0028	**
			noDep vs. Dep	t (20) = 6.605	0.0004	**
3G	cYAP/GAPDH protein fold change	One-way ANOVA		F (2, 15) = 27.26	<0.0001	***
		Tukey's multiple comparisons test	Ctrl vs. noDep	t (15) = 10.24	<0.0001	***
			Ctrl vs. Dep	t (15) = 3.365	0.0751	ns
			noDep vs. Dep	t (15) = 6.878	0.0006	**
	nYAP/H3 protein fold change	One-way ANOVA		F (2, 15) = 34.34	<0.0001	***
		Tukey's multiple comparisons test	Ctrl vs. noDep	t (15) = 7.745	0.0002	**
			Ctrl vs. Dep	t (15) = 3.744	0.0455	*
			noDep vs. Dep	t (15) = 11.49	<0.0001	***
	nYAP/cYAP protein fold change	One-way ANOVA		F (2, 15) = 14.44	0.0003	**
		Tukey's multiple comparisons test	Ctrl vs. noDep	t (15) = 3.757	0.0547	ns
			Ctrl vs. Dep	t (15) = 7.599	0.0002	**
			noDep vs. Dep	t (15) = 3.843	0.0398	*
4B	Synapse density	One-way ANOVA		F (2, 67) = 16.24	<0.0001	***
		Tukey's multiple comparisons test	Ctrl vs. noDep	t (67) = 3.727	0.0278	*
			Ctrl vs. Dep	t (67) = 8.06	<0.0001	***
			noDep vs. Dep	t (67) = 4.434	0.0071	**
	Synaptic cleft	One-way ANOVA		F (2, 67) = 8.847	0.0004	**
		Tukey's multiple comparisons test	Ctrl vs. noDep	t (67) = 1.632	0.4847	ns
			Ctrl vs. Dep	t (67) = 4.343	0.0085	**
			noDep vs. Dep	t (67) = 5.792	<0.0001	***
	PSD length	One-way ANOVA		F (2, 67) = 4.157	0.0199	*
		Tukey's multiple comparisons test	Ctrl vs. noDep	t (67) = 0.8266	0.8289	ns
			Ctrl vs. Dep	t (67) = 3.174	0.0711	ns
			noDep vs. Dep	t (67) = 3.891	0.0206	*
	PSD thickness	One-way ANOVA		F (2, 67) = 4.350	0.0167	*
		Tukey's multiple comparisons test	Ctrl vs. noDep	t (67) = 1.166	0.6891	ns
			Ctrl vs. Dep	t (67) = 3.03	0.0890	ns
			noDep vs. Dep	t (67) = 4.067	0.0147	*
4D	Mito. Density	One-way ANOVA		F (2, 67) = 10.81	<0.0001	***
		Tukey's multiple comparisons test	Ctrl vs. noDep	t (67) = 0.6884	0.8778	ns
			Ctrl vs. Dep	t (67) = 6.131	<0.0001	***
			noDep vs. Dep	t (67) = 5.379	<0.0001	***
	Mito. Volume	One-way ANOVA		F (2, 67) = 2.923	0.0465	*
		Tukey's multiple comparisons test	Ctrl vs. noDep	t (67) = 0.7644	0.8516	ns
			Ctrl vs. Dep	t (67) = 3.26	0.0619	ns
			noDep vs. Dep	t (67) = 2.488	0.1912	ns
	Mito. Length	One-way ANOVA		F (2, 67) = 10.33	<0.0001	***
		Tukey's multiple comparisons test	Ctrl vs. noDep	t (67) = 1.751	0.4351	ns
			Ctrl vs. Dep	t (67) = 6.303	<0.0001	***
			noDep vs. Dep	t (67) = 4.554	0.0055	**
	Synapse with mito.	One-way ANOVA		F (2, 67) = 1.030	0.3625	ns
	Presynpatic	Tukey's multiple comparisons test	Ctrl vs. noDep	t (67) = 0.7837	0.8447	ns
			Ctrl vs. Dep	t (67) = 2.024	0.3309	ns
			noDep vs. Dep	t (67) = 1.255	0.6499	ns
	Postsynaptic	One-way ANOVA		F (2, 67) = 6.533	0.0026	**
		Tukey's multiple comparisons test	Ctrl vs. noDep	t (67) = 1.227	0.6626	ns
			Ctrl vs. Dep	t (67) = 4.975	0.0022	**
			noDep vs. Dep	t (67) = 3.74	0.0271	*
4E	ATP production	One-way ANOVA		F (2, 21) = 23.16	<0.0001	***
		Tukey's multiple comparisons test	Ctrl vs. noDep	t (21) = 0.3225	0.9718	ns
			Ctrl vs. Dep	t (21) = 8.492	<0.0001	***
			noDep vs. Dep	t (21) = 8.169	<0.0001	***
	ETC activity	One-way ANOVA		F (2, 21) = 15.84	<0.0001	***
	Complex I	Tukey's multiple comparisons test	Ctrl vs. noDep	t (21) = 3.048	0.1028	ns
			Ctrl vs. Dep	t (21) = 4.844	0.0069	**
			noDep vs. Dep	t (21) = 7.892	<0.0001	***
	Complex II	One-way ANOVA		F (2, 21) = 8.087	0.0025	**
		Tukey's multiple comparisons test	Ctrl vs. noDep	t (21) = 1.948	0.3705	ns
			Ctrl vs. Dep	t (21) = 5.602	0.0020	**
			noDep vs. Dep	t (21) = 3.654	0.0439	*
	Complex III	One-way ANOVA		F (2, 21) = 14.46	<0.0001	***
		Tukey's multiple comparisons test	Ctrl vs. noDep	t (21) = 0.9582	0.7789	ns
			Ctrl vs. Dep	t (21) = 6.054	<0.0001	***
			noDep vs. Dep	t (21) = 7.012	<0.0001	***
	Complex IV	One-way ANOVA		F (2, 21) = 2.588	0.0989	ns
		Tukey's multiple comparisons test	Ctrl vs. noDep	t (21) = 1.523	0.5385	ns
			Ctrl vs. Dep	t (21) = 1.693	0.4677	ns
			noDep vs. Dep	t (21) = 3.216	0.0819	ns
	Complex V	One-way ANOVA		F (2, 21) = 5.723	0.0104	*
		Tukey's multiple comparisons test	Ctrl vs. noDep	t (21) = 0.9474	0.7833	ns
			Ctrl vs. Dep	t (21) = 4.535	0.0113	*
			noDep vs. Dep	t (21) = 3.588	0.0483	*
	CS activity	One-way ANOVA		F (2, 15) = 0.4576	0.6414	ns
		Tukey's multiple comparisons test	Ctrl vs. noDep	t (15) = 0.8674	0.8151	ns
			Ctrl vs. Dep	t (15) = 1.324	0.6264	ns
			noDep vs. Dep	t (15) = 2.192	0.2970	ns

4F	Cox1 mtDNA content		One-way ANOVA		F (2, 15) = 22.09	<0.0001	***
			Tukey's multiple comparisons test	Ctrl vs. noDep	t (15) = 1.995	0.3607	ns
				Ctrl vs. Dep	t (15) = 8.952	<0.0001	***
				noDep vs. Dep	t (15) = 6.958	<0.0001	***
	Cyt-b mtDNA content		One-way ANOVA		F (2, 15) = 8.859	0.0029	**
			Tukey's multiple comparisons test	Ctrl vs. noDep	t (15) = 1.809	0.4277	ns
				Ctrl vs. Dep	t (15) = 5.816	0.0025	**
				noDep vs. Dep	t (15) = 4.007	0.0318	*
4G	mRNA fold change	*PGC1-[illegible]*	One-way ANOVA		F (2, 21) = 11.54	0.0004	**
			Tukey's multiple comparisons test	Ctrl vs. noDep	t (21) = 1.413	0.4330	ns
				Ctrl vs. Dep	t (21) = 4.683	0.0004	**
				noDep vs. Dep	t (21) = 3.27	0.0109	*
		TFAM	One-way ANOVA		F (2, 21) = 10.90	0.0006	**
			Tukey's multiple comparisons test	Ctrl vs. noDep	t (21) = 0.1152	0.9963	ns
				Ctrl vs. Dep	t (21) = 5.776	0.0015	**
				noDep vs. Dep	t (21) = 5.661	0.0018	**
		Nrf1	One-way ANOVA		F (2, 21) = 3.878	0.0369	*
			Tukey's multiple comparisons test	Ctrl vs. noDep	t (21) = 2.888	0.1269	ns
				Ctrl vs. Dep	t (21) = 3.763	0.0373	*
				noDep vs. Dep	t (21) = 0.8759	0.8112	ns
		Mfn1	One-way ANOVA		F (2, 21) = 17.32	<0.0001	***
			Tukey's multiple comparisons test	Ctrl vs. noDep	t (21) = 2.352	0.2424	ns
				Ctrl vs. Dep	t (21) = 8.091	<0.0001	***
				noDep vs. Dep	t (21) = 5.739	0.0016	**
		Opa1	One-way ANOVA		F (2, 21) = 13.84	<0.0001	***
			Tukey's multiple comparisons test	Ctrl vs. noDep	t (21) = 3.676	0.0425	*
				Ctrl vs. Dep	t (21) = 7.441	<0.0001	***
				noDep vs. Dep	t (21) = 3.765	0.0372	*
		Drp1	One-way ANOVA		F (2, 21) = 8.123	0.0024	**
			Tukey's multiple comparisons test	Ctrl vs. noDep	t (21) = 1.424	0.5808	ns
				Ctrl vs. Dep	t (21) = 5.492	0.0024	**
				noDep vs. Dep	t (21) = 4.068	0.0235	*
		Fis1	One-way ANOVA		F (2, 21) = 55.34	<0.0001	***
			Tukey's multiple comparisons test	Ctrl vs. noDep	t (21) = 3.183	0.0857	ns
				Ctrl vs. Dep	t (21) = 14.18	<0.0001	***
				noDep vs. Dep	t (21) = 11	<0.0001	***
		PINK1	One-way ANOVA		F (2, 21) = 11.93	0.0003	**
			Tukey's multiple comparisons test	Ctrl vs. noDep	t (21) = 1.813	0.4206	ns
				Ctrl vs. Dep	t (21) = 6.68	0.0003	**
				noDep vs. Dep	t (21) = 4.867	0.0066	**
		Bnip3	One-way ANOVA		F (2, 21) = 10.32	0.0008	**
			Tukey's multiple comparisons test	Ctrl vs. noDep	t (21) = 3.007	0.1086	ns
				Ctrl vs. Dep	t (21) = 3.415	0.0620	ns
				noDep vs. Dep	t (21) = 6.421	<0.0001	***
		Bcl-2	One-way ANOVA		F (2, 21) = 17.63	<0.0001	***
			Tukey's multiple comparisons test	Ctrl vs. noDep	t (21) = 2.054	0.3334	ns
				Ctrl vs. Dep	t (21) = 6.025	0.0010	**
				noDep vs. Dep	t (21) = 8.079	<0.0001	***
		Bax	One-way ANOVA		F (2, 21) = 56.20	<0.0001	***
			Tukey's multiple comparisons test	Ctrl vs. noDep	t (21) = 0.3881	0.9594	ns
				Ctrl vs. Dep	t (21) = 12.79	<0.0001	***
				noDep vs. Dep	t (21) = 13.17	<0.0001	***
		VDAC2	One-way ANOVA		F (2, 21) = 9.881	0.0009	**
			Tukey's multiple comparisons test	Ctrl vs. noDep	t (21) = 1.152	0.6981	ns
				Ctrl vs. Dep	t (21) = 5.929	0.0011	**
				noDep vs. Dep	t (21) = 4.776	0.0077	**
		Cyc-s	One-way ANOVA		F (2, 21) = 19.49	<0.0001	***
			Tukey's multiple comparisons test	Ctrl vs. noDep	t (21) = 1.768	0.4381	ns
				Ctrl vs. Dep	t (21) = 8.376	<0.0001	***
				noDep vs. Dep	t (21) = 6.609	<0.0001	***
		GAPDH	One-way ANOVA		F (2, 21) = 0.6495	0.5325	ns
			Tukey's multiple comparisons test	Ctrl vs. noDep	t (21) = 0.1719	0.9975	ns
				Ctrl vs. Dep	t (21) = 0.8898	0.7658	ns
				noDep vs. Dep	t (21) = 1.062	0.6576	ns
4H	Protein fold change	*PGC-1a*	One-way ANOVA		F (2, 16) = 13.58	0.0004	**
			Tukey's multiple comparisons test	Ctrl vs. noDep	t (16) = 0.995	0.7648	ns
				Ctrl vs. Dep	t (16) = 6.938	0.0004	**
				noDep vs. Dep	t (16) = 5.727	0.0025	**
		Mfn1	One-way ANOVA		F (2, 16) = 8.451	0.0031	**
			Tukey's multiple comparisons test	Ctrl vs. noDep	t (16) = 1.676	0.4786	ns
				Ctrl vs. Dep	t (16) = 5.713	0.0026	**
				noDep vs. Dep	t (16) = 3.89	0.0359	*
		Drp1	One-way ANOVA		F (2, 16) = 7.383	0.0054	**
			Tukey's multiple comparisons test	Ctrl vs. noDep	t (16) = 0.8533	0.8203	ns
				Ctrl vs. Dep	t (16) = 5.155	0.0058	**
				noDep vs. Dep	t (16) = 4.145	0.0251	*
		VDAC2	One-way ANOVA		F (2, 16) = 15.49	0.0002	**
			Tukey's multiple comparisons test	Ctrl vs. noDep	t (16) = 1.907	0.3903	ns
				Ctrl vs. Dep	t (16) = 5.894	0.0020	**
				noDep vs. Dep	t (16) = 7.517	<0.0001	**
5B	mRNA fold change	*YAP*	Unpaired t test	si-NC vs. si-YAP	t (10) = 8.426	<0.0001	***
		PGC-1a			t (10) = 5.621	<0.0001	***
		TFAM			t (10) = 8.466	<0.0001	***
		Mfn1			t (10) = 6.817	<0.0001	***
		Opa1			t (10) = 7.824	<0.0001	***
		Drp1			t (10) = 3.98	0.0026	**
		VDAC2			t (10) = 3.109	0.0111	*
		Cyc-s			t (10) = 2.352	0.0405	*
		GAPDH			t (10) = 0.246	0.6685	ns
5C	Protein fold change	*YAP*	Unpaired t test	si-NC vs. si-YAP	t (10) = 4.471	<0.0001	***
		PGC-1a			t (10) = 3.203	<0.0001	***
		TFAM			t (10) = 4.968	<0.0001	***
		VDAC2			t (10) = 2.487	0.0321	*

5D	JC1 R/G ratio	Unpaired t test	si-NC vs. si-YAP	t (18) = 5.809	<0.0001	***
5E	ATP production	Unpaired t test	si-NC vs. si-YAP	t (14) = 6.237	<0.0001	***
	ETC activity *Complex I*			t (18) = 4.208	<0.0001	***
	Complex II			t (14) = 3.472	0.0037	**
	Complex V			t (14) = 2.563	0.0225	*
5F	mtDNA content *Cox*	Unpaired t test	si-NC vs. si-YAP	t (14) = 7.550	<0.0001	***
	Cyt-b			t (14) = 4.874	<0.0001	***
5I	TUNEL+ cells	Unpaired t test	NC vs. ko-YAP	t (14) = 8.940	<0.0001	***
5J	SPT - Sucorese preference	Two-way ANOVA	Interaction	F (1, 36) = 6.529	0.0150	*
			Stress	F (1, 36) = 4.105	0.0402	*
			ko-YAP	F (1, 36) = 14.47	0.0005	**
		Šídák's multiple comparisons test	No stress NC vs. ko-YAP	t (36) = 0.8062	0.6699	ns
			Stressed	t (36) = 5.028	<0.0001	***
	EPM - Open arm time	Two-way ANOVA	Interaction	F (1, 36) = 3.032	0.0902	ns
			Stress	F (1, 36) = 8.195	0.0070	**
			ko-YAP	F (1, 36) = 7.201	0.0109	*
		Šídák's multiple comparisons test	No stress NC vs. ko-YAP	t (36) = 0.6082	0.7947	ns
			Stressed	t (36) = 3.498	0.0025	**
	FST - Immobility time	Two-way ANOVA	Interaction	F (1, 36) = 2.198	0.1469	ns
			Stress	F (1, 36) = 38.83	<0.0001	***
			ko-YAP	F (1, 36) = 6.168	0.0178	*
		Šídák's multiple comparisons test	No stress NC vs. ko-YAP	t (36) = 0.6461	0.7718	ns
			Stressed	t (36) = 3.136	0.0068	**
	NSF - Feeding latency	Two-way ANOVA	Interaction	F (1, 36) = 16.12	0.0003	**
			Stress	F (1, 36) = 11.19	0.0019	**
			ko-YAP	F (1, 36) = 14.35	0.0006	**
		Šídák's multiple comparisons test	No stress NC vs. ko-YAP	t (36) = 0.1466	0.9866	ns
			Stressed	t (36) = 6.169	<0.0001	***
6B	SPT - Sucorese preference	Unpaired t test	Scr vs. oe-YAP	t (38) = 2.042	0.0481	*
	SPT - Total fluid intake			t (38) = 1.211	0.2332	ns
	EPM - Open arm time	Unpaired t test	Scr vs. oe-YAP	t (38) = 3.445	0.0014	**
	EPM - Open arm entries			t (38) = 2.289	0.0278	*
	FST - Immobility time	Unpaired t test	Scr vs. oe-YAP	t (38) = 2.0474	0.0449	*
	FST - Immobility latency			t (38) = 7.145	<0.0001	***
6E	NSF - Feeding latency	Two-way ANOVA	Interaction	F (1, 25) = 0.05028	0.8244	ns
			Dep	F (1, 25) = 0.3952	0.5353	ns
			oe-YAP	F (1, 25) = 21.18	<0.0001	***
		Šídák's multiple comparisons test	Scr noDep vs. Dep	t (25) = 3.693	0.0022	**
			oe-YAP	t (25) = 2.997	0.0121	*
7B	SPT - Sucorese preference	Two-way ANOVA	Interaction	F (1, 48) = 10.21	0.0025	**
			Dep	F (1, 48) = 24.78	<0.0001	***
			XMU	F (1, 48) = 1.947	0.1694	ns
		Šídák's multiple comparisons test	noDep DMSO vs. XMU	t (48) = 1.384	0.3155	ns
			Dep	t (48) = 3.022	0.0080	**
	SPT - Total fluid intake	Two-way ANOVA	Interaction	F (1, 48) = 0.3506	0.5566	ns
			Dep	F (1, 48) = 12.38	0.0010	**
			XMU	F (1, 48) = 4.240	0.0451	*
		Šídák's multiple comparisons test	noDep DMSO vs. XMU	t (48) = 1.144	0.4502	ns
			Dep	t (48) = 1.728	0.1731	ns
	EPM - Open arm time	Two-way ANOVA	Interaction	F (1, 48) = 12.75	0.0008	**
			Dep	F (1, 48) = 27.04	<0.0001	***
			XMU	F (1, 48) = 4.699	0.0352	*
		Šídák's multiple comparisons test	noDep DMSO vs. XMU	t (48) = 1.079	0.4902	ns
			Dep	t (48) = 3.778	<0.0001	***
	EPM - Open arm entries	Two-way ANOVA	Interaction	F (1, 48) = 5.527	0.0229	*
			Dep	F (1, 48) = 9.526	0.0034	**
			XMU	F (1, 48) = 2.229	0.1420	ns
		Šídák's multiple comparisons test	noDep DMSO vs. XMU	t (48) = 0.6595	0.7626	ns
			Dep	t (48) = 2.531	0.0292	*
	FST - Immobility time	Two-way ANOVA	Interaction	F (1, 48) = 4.337	0.0426	*
			Dep	F (1, 48) = 12.41	0.0009	**
			XMU	F (1, 48) = 5.815	0.0198	*
		Šídák's multiple comparisons test	noDep DMSO vs. XMU	t (48) = 0.2528	0.9606	ns
			Dep	t (48) = 2.958	<0.0001	***
	FST - Immobility latency	Two-way ANOVA	Interaction	F (1, 48) = 7.795	0.0075	**
			Dep	F (1, 48) = 89.22	<0.0001	***
			XMU	F (1, 48) = 0.001419	0.9701	ns
		Šídák's multiple comparisons test	noDep DMSO vs. XMU	t (48) = 2.117	0.0773	ns
			Dep	t (48) = 1.863	0.1325	ns
7D	cFos+ cells	Two-way ANOVA	Interaction	F (1, 28) = 3.356	0.0776	ns
			Dep	F (1, 28) = 13.06	0.0012	**
			XMU	F (1, 28) = 3.131	0.0877	ns
		Šídák's multiple comparisons test	noDep DMSO vs. XMU	t (28) = 0.04408	0.9998	ns
			Dep	t (48) = 2.547	0.0331	*
7F	Synapse density	Two-way ANOVA	Interaction	F (1, 32) = 0.1569	0.6946	ns
			Dep	F (1, 32) = 16.87	0.0003	**
			XMU	F (1, 32) = 11.55	0.0018	**
		Šídák's multiple comparisons test	noDep DMSO vs. XMU	t (32) = 2.123	0.0815	ns
			Dep	t (32) = 2.683	0.0228	*
	PSD length	Two-way ANOVA	Interaction	F (1, 32) = 2.658	0.1128	ns
			Dep	F (1, 32) = 4.715	0.0374	*
			XMU	F (1, 32) = 4.925	0.0337	*
		Šídák's multiple comparisons test	noDep DMSO vs. XMU	t (32) = 0.4164	0.8975	ns
			Dep	t (32) = 2.722	0.0207	*
	PSD thickness	Two-way ANOVA	Interaction	F (1, 32) = 2.876	0.0996	ns
			Dep	F (1, 32) = 15.60	0.0004	**
			XMU	F (1, 32) = 3.300	0.0786	ns
		Šídák's multiple comparisons test	noDep DMSO vs. XMU	t (32) = 0.08551	0.9954	ns
			Dep	t (32) = 2.484	0.0365	*
7G	Mito. density	Two-way ANOVA	Interaction	F (1, 32) = 0.7529	0.3920	ns
			Dep	F (1, 32) = 6.198	0.0182	*
			XMU	F (1, 32) = 8.102	0.0077	**
		Šídák's multiple comparisons test	noDep DMSO vs. XMU	t (32) = 1.399	0.3134	ns

				Dep	t (32) = 2.626	0.0261	*
	Mito. length		Two-way ANOVA	Interaction	F (1, 32) = 4.622	0.0392	*
				Dep	F (1, 32) = 8.295	0.0070	**
				XMU	F (1, 32) = 40.05	<0.0001	***
			Šídák's multiple comparisons test	noDep DMSO vs. XMU	t (32) = 2.954	0.0116	*
				Dep	t (32) = 5.995	<0.0001	***
7H	Protein fold change	*pLats1/Lats*	Two-way ANOVA	Interaction	F (1, 20) = 1.071	0.3131	ns
				Dep	F (1, 20) = 35.02	<0.0001	***
				XMU	F (1, 20) = 105.1	<0.0001	***
			Šídák's multiple comparisons test	noDep DMSO vs. XMU	t (20) = 6.517	<0.0001	***
				Dep	t (20) = 7.98	<0.0001	***
		pYAP/YAP	Two-way ANOVA	Interaction	F (1, 20) = 5.146	0.0345	*
				Dep	F (1, 20) = 44.55	<0.0001	***
				XMU	F (1, 20) = 81.82	<0.0001	***
			Šídák's multiple comparisons test	noDep DMSO vs. XMU	t (20) = 4.792	<0.0001	***
				Dep	t (20) = 8.000	<0.0001	***
7I	ATP production		Two-way ANOVA	Interaction	F (1, 20) = 2.710	0.1153	ns
				Dep	F (1, 20) = 10.78	0.0037	**
				XMU	F (1, 20) = 29.29	<0.0001	***
			Šídák's multiple comparisons test	noDep DMSO vs. XMU	t (20) = 2.663	0.0297	*
				Dep	t (20) = 4.991	<0.0001	***
	ETX complex I activity		Two-way ANOVA	Interaction	F (1, 20) = 0.09311	0.7634	ns
				Dep	F (1, 20) = 18.25	0.0004	**
				XMU	F (1, 20) = 11.14	0.0033	**
			Šídák's multiple comparisons test	noDep DMSO vs. XMU	t (20) = 2.144	0.0870	ns
				Dep	t (20) = 2.576	0.0358	*
	mtDNA content	*Cox1*	Two-way ANOVA	Interaction	F (1, 20) = 2.039	0.1688	ns
				Dep	F (1, 20) = 26.98	<0.0001	***
				XMU	F (1, 20) = 26.13	<0.0001	***
			Šídák's multiple comparisons test	noDep DMSO vs. XMU	t (20) = 2.605	0.0336	*
				Dep	t (20) = 4.624	<0.0001	***
		Cyt-b	Two-way ANOVA	Interaction	F (1, 20) = 2.138	0.1592	ns
				Dep	F (1, 20) = 6.132	0.0223	*
				XMU	F (1, 20) = 10.15	0.0046	**
			Šídák's multiple comparisons test	noDep DMSO vs. XMU	t (20) = 1.219	0.4178	ns
				Dep	t (20) = 3.287	0.0074	**
7K	SPT - Sucorese preference		One-way ANOVA		F (2, 33) = 8.424	0.0011	**
			Tukey's multiple comparisons test	NC+DMSO vs. cYAP+DMSO	t (33) = 5.377	0.0017	**
				NC+DMSO vs. cYAP+XMU	t (33) = 4.582	0.0075	**
				cYAP+DMSO vs. cYAP+XMU	t (33) = 0.795	0.8410	ns
	SPT - Total fluid intake		One-way ANOVA		F (2, 33) = 3.236	0.0521	ns
			Tukey's multiple comparisons test	NC+DMSO vs. cYAP+DMSO	t (33) = 0.5363	0.9240	ns
				NC+DMSO vs. cYAP+XMU	t (33) = 3.349	0.0603	ns
				cYAP+DMSO vs. cYAP+XMU	t (33) = 2.813	0.1308	ns
	EPM - Open arm time		One-way ANOVA		F (2, 33) = 4.882	0.0139	*
			Tukey's multiple comparisons test	NC+DMSO vs. cYAP+DMSO	t (33) = 3.947	0.0230	*
				NC+DMSO vs. cYAP+XMU	t (33) = 3.695	0.0349	*
				cYAP+DMSO vs. cYAP+XMU	t (33) = 0.2519	0.9827	ns
	EPM - Open arm entries		One-way ANOVA		F (2, 33) = 7.679	0.0018	**
			Tukey's multiple comparisons test	NC+DMSO vs. cYAP+DMSO	t (33) = 5.231	0.0022	**
				NC+DMSO vs. cYAP+XMU	t (33) = 4.218	0.0144	*
				cYAP+DMSO vs. cYAP+XMU	t (33) = 1.012	0.7559	ns
	FST - Immobility time		One-way ANOVA		F (2, 33) = 11.98	<0.0001	***
			Tukey's multiple comparisons test	NC+DMSO vs. cYAP+DMSO	t (33) = 6.861	<0.0001	***
				NC+DMSO vs. cYAP+XMU	t (33) = 4.224	0.0142	*
				cYAP+DMSO vs. cYAP+XMU	t (33) = 2.637	0.1650	ns
	FST - Immobility latency		One-way ANOVA		F (2, 33) = 14.53	<0.0001	***
			Tukey's multiple comparisons test	NC+DMSO vs. cYAP+DMSO	t (33) = 7.509	<0.0001	***
				NC+DMSO vs. cYAP+XMU	t (33) = 4.889	0.0042	**
				cYAP+DMSO vs. cYAP+XMU	t (33) = 2.62	0.1686	ns
7J	OFT - Total distance		One-way ANOVA		F (2, 33) = 1.016	0.3732	ns
			Tukey's multiple comparisons test	NC+DMSO vs. cYAP+DMSO	t (33) = 2.015	0.3399	ns
				NC+DMSO vs. cYAP+XMU	t (33) = 1.035	0.7466	ns
				cYAP+DMSO vs. cYAP+XMU	t (33) = 0.9806	0.7690	ns
8B	mRNA fold change	*14-3-3(*	One-way ANOVA		F (2, 14) = 1.408	0.2773	ns
		14-3-3Z	One-way ANOVA		F (2, 14) = 7.086	0.0075	**
			Tukey's multiple comparisons test	Ctrl vs. noDep	t (14) = 4.516	0.0168	*
				Ctrl vs. Dep	t (14) = 5.007	0.0086	**
				noDep vs. Dep	t (14) = 0.4011	0.9568	ns
		14-3-3H	One-way ANOVA		F (2, 14) = 27.10	<0.0001	***
			Tukey's multiple comparisons test	Ctrl vs. noDep	t (14) = 2.174	0.3045	ns
				Ctrl vs. Dep	t (14) = 6.663	<0.0001	***
				noDep vs. Dep	t (14) = 10.03	<0.0001	***
		14-3-3E	One-way ANOVA		F (2, 14) = 3.737	0.0491	*
			Tukey's multiple comparisons test	Ctrl vs. noDep	t (14) = 1.556	0.5293	ns
				Ctrl vs. Dep	t (14) = 3.744	0.0473	*
				noDep vs. Dep	t (14) = 2.412	0.2376	ns
		14-3-3B	One-way ANOVA		F (2, 14) = 0.1335	0.8762	ns
		14-3-3Q	One-way ANOVA		F (2, 14) = 1.611	0.2346	ns
		14-3-3S	One-way ANOVA		F (2, 14) = 1.995	0.1729	ns
8C	Protein fold change	*14-3-3I*	One-way ANOVA		F (2, 15) = 9.336	0.0023	**
			Tukey's multiple comparisons test	Ctrl vs. noDep	t (15) = 0.9986	0.7636	ns
				Ctrl vs. Dep	t (15) = 5.720	0.0029	**
				noDep vs. Dep	t (15) = 4.722	0.0117	*
8H	Total YAP intensity		Unpaired t test	DMSO vs. BV02	t (78) = 0.8813	0.3809	ns
	Nuclear YAP intensity				t (78) = 13.57	<0.0001	***
8J	Nuclear YAP protein		Unpaired t test	si-NC vs. si-14-3-3h	t (14) = 5.992	<0.0001	***
	Total YAP protein				t (14) = 2.098	0.0543	ns
8L	SPT - Sucorese preference		Two-way RM ANOVA	Interaction	F (1, 15) = 8.966	0.0091	**
				Time	F (1, 15) = 1.936	0.1844	ns
				sh-14-3-3h	F (1, 15) = 30.56	<0.0001	***
			Šídák's multiple comparisons test	NC: Before vs. After	t (15) = 1.741	0.1937	ns
				sh-14-3-3h: Before vs. After	t (15) = 6.212	<0.0001	***

	EPM - Open arm time		Two-way RM ANOVA	Interaction		F (1, 15) = 1.520	0.2366	ns
				Time		F (1, 15) = 2.386	0.1433	ns
				sh-14-3-3h		F (1, 15) = 4.340	0.0547	ns
			Šídák's multiple comparisons test	NC:	Before vs. After	t (15) = 0.5845	0.813	ns
				sh-14-3-3h:	Before vs. After	t (15) = 2.417	0.0569	ns
	FST - Immobility time		Two-way RM ANOVA	Interaction		F (1, 15) = 8.899	0.0093	**
				Time		F (1, 15) = 0.5015	0.4897	ns
				sh-14-3-3h		F (1, 15) = 31.02	<0.0001	***
			Šídák's multiple comparisons test	NC:	Before vs. After	t (15) = 1.777	0.1824	ns
				sh-14-3-3h:	Before vs. After	t (15) = 6.234	<0.0001	***
	NSF - Feeding latency		Two-way RM ANOVA	Interaction		F (1, 15) = 13.25	0.0024	**
				Time		F (1, 15) = 4.402	0.0432	*
				sh-14-3-3h		F (1, 15) = 1.528	0.2355	ns
			Šídák's multiple comparisons test	NC:	Before vs. After	t (15) = 1.652	0.2243	ns
				sh-14-3-3h:	Before vs. After	t (15) = 3.554	0.0058	**
8M	OFT - Total distance		Two-way RM ANOVA	Interaction		F (1, 15) = 1.290	0.2739	ns
				Time		F (1, 15) = 0.01574	0.9018	ns
				sh-14-3-3h		F (1, 15) = 2.552	0.1310	ns
8N	mRNA fold change	*PGC-1α*	Unpaired t test	NC vs. sh-14-3-3h		t (14) = 5.616	<0.0001	***
		TFAM				t (14) = 3.327	0.0050	**
		Mfn1				t (14) = 2.738	0.0160	*
		Opa1				t (14) = 1.060	0.3071	ns
		Drp1				t (14) = 3.145	0.0072	**
		VDAC2				t (14) = 6.471	<0.0001	***
8O	ATP production		Unpaired t test	NC vs. sh-14-3-3h		t (14) = 2.555	0.0229	
	ETC activity	*Complex I*				t (14) = 3.423	0.0041	
		Complex II				t (14) = 4.707	0.0003	
		Complex III				t (14) = 6.458	<0.0001	
		Complex V				t (14) = 2.163	0.0483	
	CS activity					t (14) = 1.227	0.2402	
	mtDNA content	*Cox1*				t (14) = 2.865	0.0125	
		Cyt-b				t (14) = 5.706	<0.0001	
