# Supplementary material for: Chronic stress dysregulates the Hippo/YAP/14-3-3η pathway and induces mitochondrial damage in basolateral amygdala in a mouse model of depression
Source: Theranostics. 2024 Jun 11;14(9):3653–73. doi: 10.7150/thno.92676 (PMC11209716; doi:10.7150/thno.92676)
Supplement: Supplementary file 1 — Supplementary data, figures and tables. [file thnov14p3653s1.zip › Supplementary Figures.pdf]

SUPPLEMENTARY FIUGURES

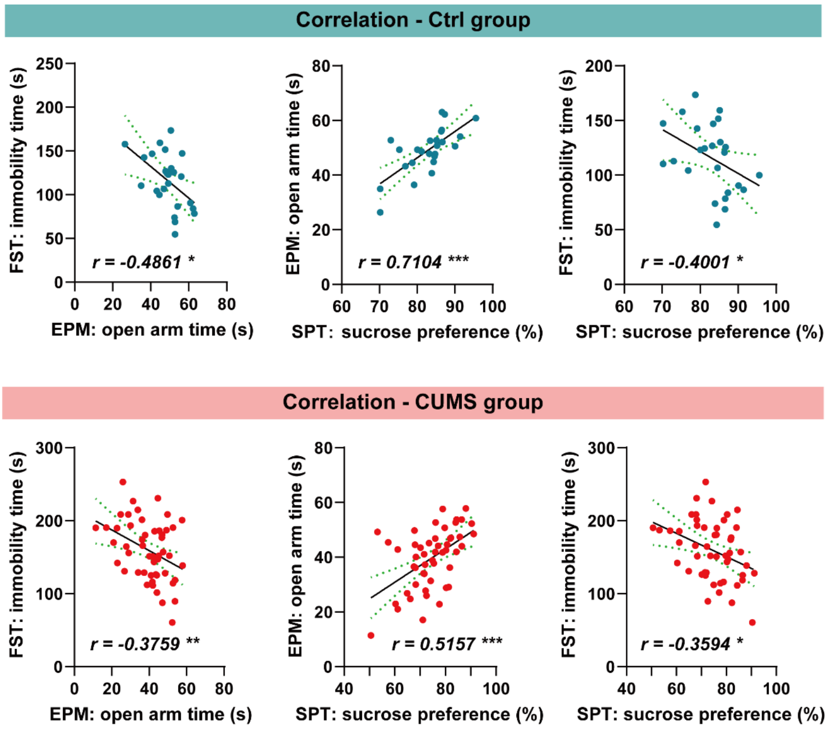

Figure S1. Correlation analysis of sucrose preference, open-arm time, and immobility time in mice.

Correlation analysis of the three measures for the assessment of depressive-like phenotypes in Ctrl and CUMS-treated mice.

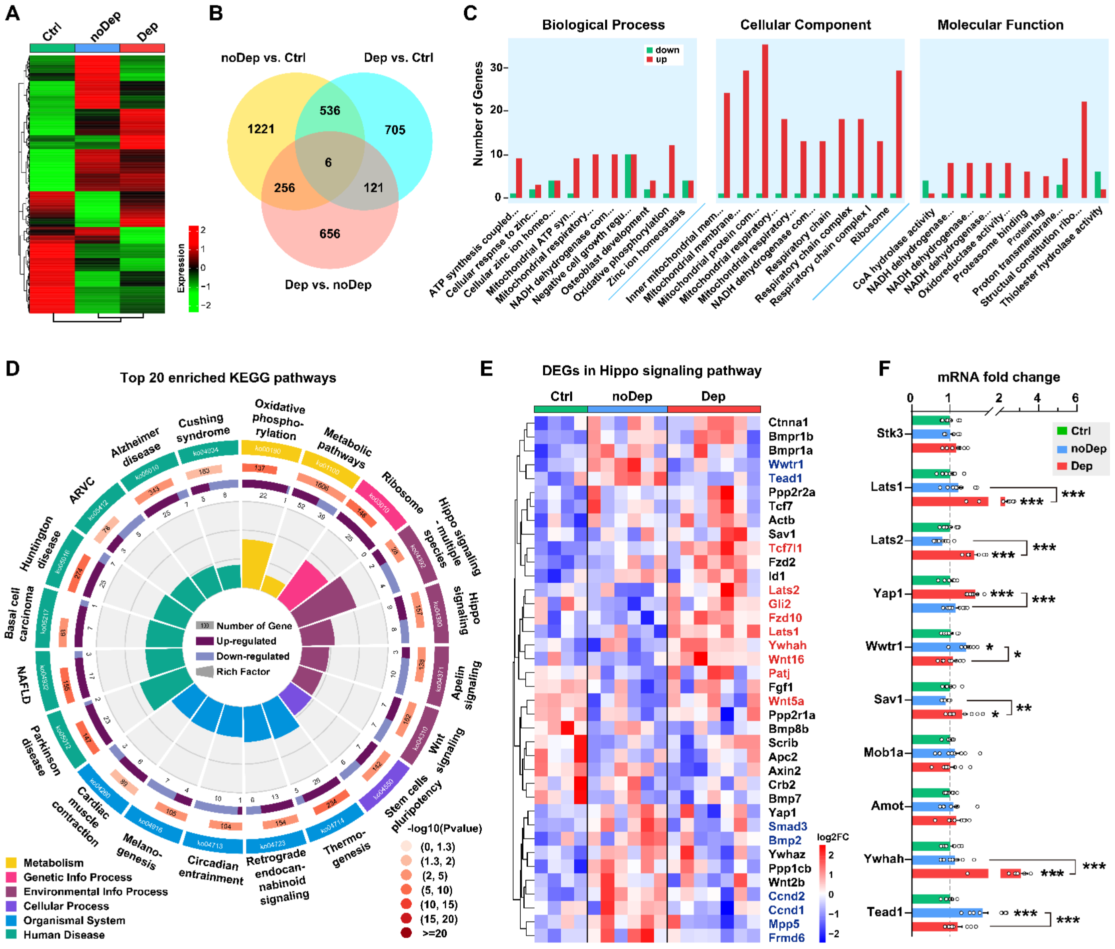

Figure S2. Enrichment analysis of BLA transcriptome data in mice.

A, Volcano plot diagrams of DEGs between Ctrl, noDep, and Dep group. Red and green dots represent significantly upregulated/downregulated genes. **B-D,** Top 20 pathways in KEGG s enrichment analysis. Comparisons were made between Ctrl, noDep, and Dep group, respectively. **E,** Diagram of Hippo signaling pathway. Red and green genes represent upregulated/downregulated genes in noDep vs. Dep group.

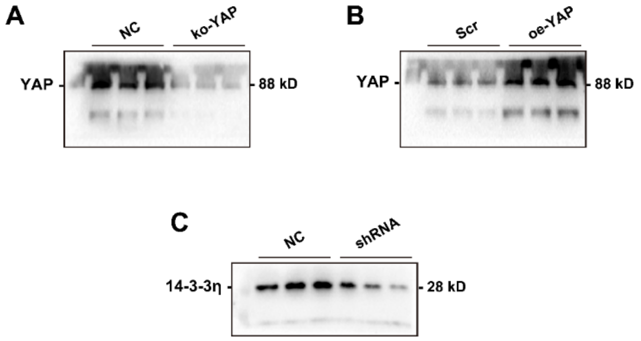

Figure S3. Immunoblots data validating knockdown or overexpression of YAP or 14-3-3η.

A, AAV-mediated YAP knockout in BLA of Yap1 floxed mice. A total of 20 μg protein was loaded in each lane. **B,** AAV-mediated YAP overexpression in BLA of C57 wildtype mice. A total of 15 μg protein was loaded in each lane. **C,** shRNA-induced YAP knockdown in BLA of C57 wildtype mice. A total of 20 μg protein was loaded in each lane.

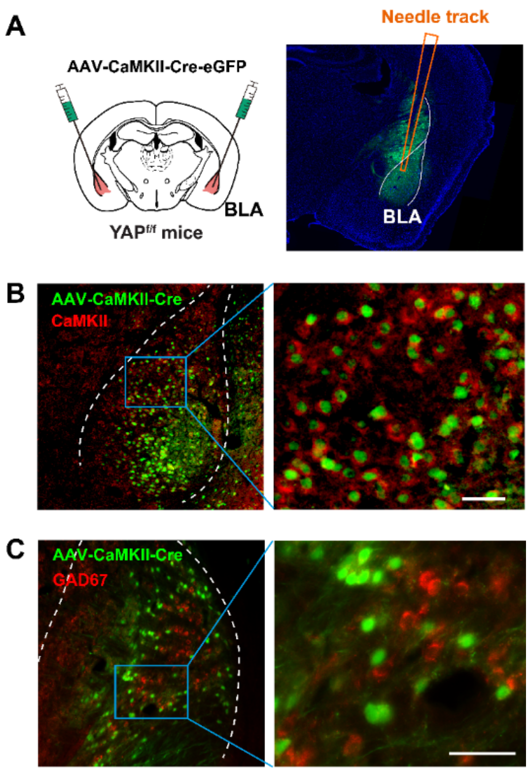

Figure S4. Validation of the specificity of the virus in BLA.

A, AAV-mediated knockout of YAP in BLA glutamatergic neurons from Yap1-floxed mice. The virus was injected into bilateral BLA and allowed to express for 1-3 weeks, and then the mice were sacrificed for brain sections and immunofluorescence staining. The fluorescent image illustrates the needle track and injection site. Note that due to the high intensity of GFP expressed by the virus, green fluorescence penetrates and interferes with red fluorescence of CaMKII and GAD67 antibodies, resulting in poor image quality. We adjusted the injection volume of the virus and shortened its expression time appropriately to optimize the double labeled images. **B,** Virus (green) was co-labeled with CaMKII antibody (red). 120 nL of virus was injected into bilateral BLA and allowed to express for 3 weeks. Scale bar = 50 μm. **C,** Virus (green) was co-labeled with GAD67 antibody (red). 90 nL of virus was injected into bilateral BLA and allowed to express for 1 weeks. Scale bar = 50 μm.

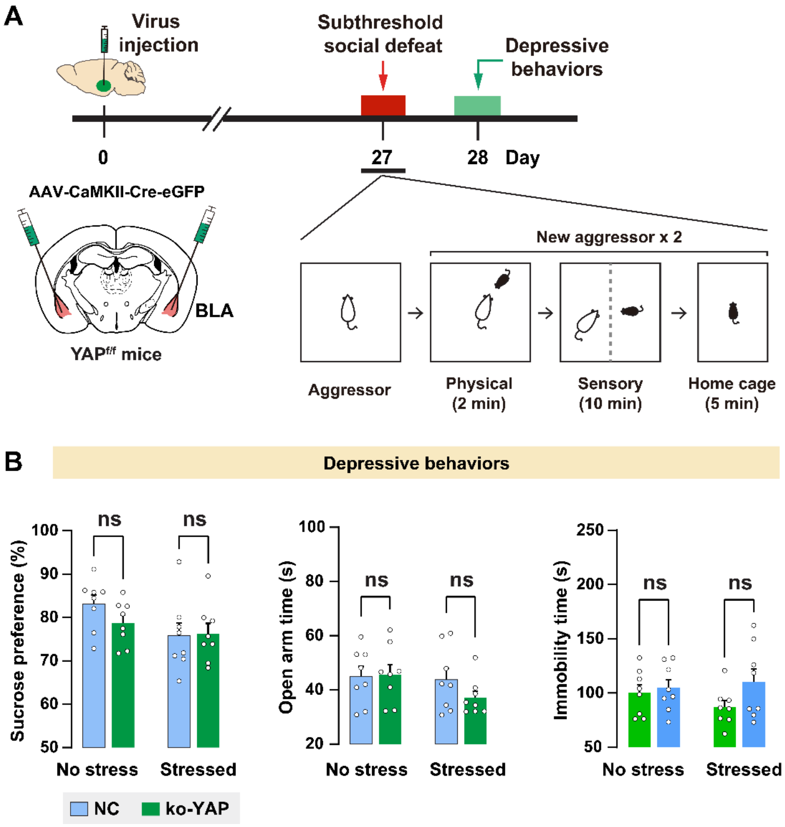

Figure S5. Subthreshold social defeat stress failed to trigger depressive-like behavior in ko-YAP mice.

A, Schematic diagram of the experiment. The one-day subthreshold social defeat stress (SSDS) protocol was used following the previous report by Chaudhury et al. (*Nature*, 2013). **B,** Depressive-like behaviors in NC and ko-YAP mice subjected to the subthreshold paradigm. n = 8 mice/group.

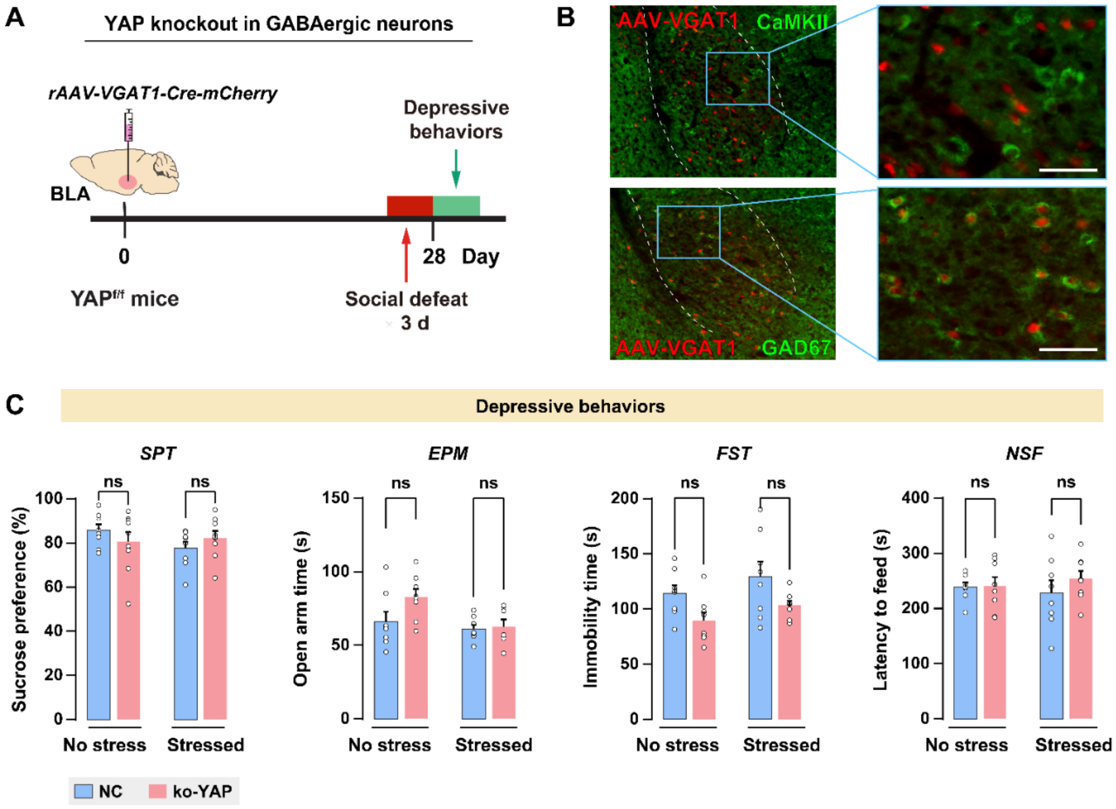

Figure S6. Yap knockout in BLA GABAergic neurons failed to affect depressive-like behaviors.

A, Schematic diagram of the experiment. Yap1-floxed mice were injected with virus into bilateral BLA and allowed to express for 4 weeks, and then the mice were subjected to social defeat stress and were tested for depressive behaviors. **B,** Representative immunofluorescent images validating AAV-mediated knockout of YAP in BLA GABAergic neurons from Yap1-floxed mice. Scale bar = 50 μm. **C,** Depressive-like behaviors in NC and ko-YAP mice following the 3-day social defeat stress. n = 8 mice/group. The result showed that Yap knockout in BLA GABAergic neurons has no significant effect on the depressive-like phenotype.
