# Supplementary material for: Chronic stress dysregulates the Hippo/YAP/14-3-3η pathway and induces mitochondrial damage in basolateral amygdala in a mouse model of depression
Source: Theranostics. 2024 Jun 11;14(9):3653–73. doi: 10.7150/thno.92676 (PMC11209716; doi:10.7150/thno.92676)
Supplement: Supplementary file 1 — Supplementary data, figures and tables. [file thnov14p3653s1.zip › Supplementary Table S2.docx]

**Table S2** The PCR primers used in this study.

| **Gene** | **Sequence (5' → 3')** |
| --- | --- |
| *Gapdh* | F: ACCCAGAAGACTGTGGATGG  R: TCATCCAAATACTCCACACGC |
| *Stk3 (MST2)* | F: GTTGACAAACCGTCTTATGGAAG  R: CATATGGACTGTCACATTGTTGC |
| *Lats1* | F: AGTGCTACTGCGAACAGGATA  R: GGAGGTTGTCCCACCAACAT |
| *Lats2* | F: GTTTCCAACTGTCGCTGTGG  R: AGAGCTGCGTGTACCCTTT |
| *Yap1* | F: ATTTCGGCAGGCAATACGGA  R: TGCGCAGAGCTAATTCCTGA |
| *Wwtr1 (TAZ)* | F: CGGTTCCGGGGATAAAGATGA  R: GGCTTGGGGTTCATGACAGA |
| *Sav1* | F: GGGAGGCACACTTCAGGTATT  R: GCCAGGAGGAAGGGGTAAAT |
| *Mob1a* | F: GCATCATTAGAAGAATCAGCAGCC  R: CCTCAGATTGCCACTTCCGA |
| *Amot* | F: GGCTGCACAGAGAGACACAA  R: CTTCCATGTCCAAGCAACGC |
| *Tead1* | F: GAGCGACTCGGCAGATAAGC  R: CCACACGGCGGATAGATAGC |
| *Cox1* | F: GCCTTTCAGGAATACCACGA  R: AGGTTGGTTCCTCGAATGTG |
| *Cytb* | F: ATTCCTTCATGTCGGACGAG  R: ACTGAGAAGGCCCCCTCAAAT |
| *Ppargc1a (PGC-1α)* | F: TATGGAGTGACATAGAGTGTGCT  R: CCACTTCAATCCACCCAGAAAG |
| *Tfam* | F: GGAATGTGGAGCGTGCTAAAA  R: ACAAGACTGATAGACGAGGGG |
| *Nrf1* | F: TATGGCGGAAGTAATGAAAGACG  R: CAACGTAAGCTCTGCCTTGTT |
| *Mfn1* | F: CCTACTGCTCCTTCTAACCCA  R: AGGGACGCCAATCCTGTGA |
| *Opa1* | F: CGACTTTGCCGAGGATAGCTT  R: CGTTGTGAACACACTGCTCTTG |
| *Dnm1 (Drp1)* | F: TTACGGTTCCCTAAACTTCACG  R: GTCACGGGCAACCTTTTACGA |
| *Fis1* | F: TGTCCAAGAGCACGCAATTTG  R: CCTCGCACATACTTTAGAGCCTT |
| *Pink1* | F: TTCTTCCGCCAGTCGGTAG  R: CTGCTTCTCCTCGATCAGCC |
| *Bnip3* | F: TCCTGGGTAGAACTGCACTTC  R: GCTGGGCATCCAACAGTATTT |
| *Bcl2* | F: ATGCCTTTGTGGAACTATATGGC  R: GGTATGCACCCAGAGTGATGC |
| *Bax* | F: TGAAGACAGGGGCCTTTTTG  R: AATTCGCCGGAGACACTCG |
| *Vdac2* | F: CTCCACCCTATGCTGACCTC  R: CCCGCTAACTTTACCAGTGTCT |
| *Cycs* | F: CCAAATCTCCACGGTCTGTTC  R: ATCAGGGTATCCTCTCCCCAG |
| *Ywhag*  *(14-3-3 gamma)* | F: GTGACCGAGCTGAACGAAC  R: GATGCTGCTGATGACCCTCC |
| *Ywhaz*  *(14-3-3 zeta)* | F: GAAAAGTTCTTGATCCCCAATGC  R: TGTGACTGGTCCACAATTCCTT |
| *Ywhah*  *(14-3-3 eta)* | F: ACGAAGATCGAAATCTCCTCTCT  R: CCGGTAGGCTTTAACTTTCTCCA |
| *Ywhae*  *(14-3-3 epsilon)* | F: ACTGGCGAGTCCAAGGTTTTC  R: TGTCATCGCAATGTCACTAGC |
| *Ywhab*  *(14-3-3 beta)* | F: TGGATAAGAGTGAGCTGGTACA  R: CGTGTCCCTGCTCTGTTACG |
| *Ywhaq*  *(14-3-3 theta)* | F: ATTGAGCAGAAGACCGACACC  R: TGTTTTCGATCATCGCCACAA |
| *Ywhas*  *(14-3-3 sigma/Sfn)* | F: GTGTGTGCGACACCGTACT  R: CTCGGCTAGGTAGCGGTAG |
