# Supplementary material for: Chronic stress dysregulates the Hippo/YAP/14-3-3η pathway and induces mitochondrial damage in basolateral amygdala in a mouse model of depression
Source: Theranostics. 2024 Jun 11;14(9):3653–73. doi: 10.7150/thno.92676 (PMC11209716; doi:10.7150/thno.92676)
Supplement: Supplementary file 1 — Supplementary data, figures and tables. [file thnov14p3653s1.zip › Supplementary Table S1.docx]

**Table S1** Antibodies used for Western blotting.

| **Protein** | **Supplier** | **Lot number** | **RRID**s | **Concentration** | **Mw (kDa)** |
| --- | --- | --- | --- | --- | --- |
| Lats1 | Abcam | ab70561 | AB_1209494 | 1:2000 | 160 |
| Phospho-Lats1(Thr1079) | CST | #8654 | AB_10971635 | 1:1000 | 140 |
| TEAD1 | CST | #12292 | AB_2797873 | 1:1000 | 50 |
| YAP | CST | #4912 | AB_2218911 | 1:1000 | 88 |
| Phospho-YAP (Ser127) | CST | #4911 | AB_2218913 | 1:1000 | 88 |
| GAPDH | CST | #5174 | AB_10622025 | 1:1000 | 37 |
| Histone H3 | CST | #4499 | AB_10544537 | 1:2000 | 17 |
| PGC-1α | Proteintech | #66369-1 | AB_2828002 | 1:1000 | 100 |
| Mfn1 | Abcam | ab221661 | AB_2941083 | 1:1000 | 84 |
| Drp1 | CST | # 8570 | AB_10950498 | 1:1000 | 78 |
| VDAC2 | CST | #9412 | AB_10828727 | 1:1000 | 30 |
| TFAM | Abcam | ab138351 | AB_2651017 | 1:2000 | 29 |
| 14-3-3η | CST | #9640 | AB_2218073 | 1:1000 | 28 |
| Flag tag | CST | #14793 | AB_2572291 | 1:1000 | / |
